# Photocontrolled multiple-state photochromic benzo[*b*]phosphole thieno[3,2*-b*]phosphole-containing alkynylgold(I) complex via selective light irradiation

**DOI:** 10.1038/s41467-021-27711-9

**Published:** 2022-01-10

**Authors:** Nathan Man-Wai Wu, Maggie Ng, Vivian Wing-Wah Yam

**Affiliations:** Institute of Molecular Functional Materials, State Key Laboratory of Synthetic Chemistry and Department of Chemistry, The University of Hong Kong, Pokfulam Road, Hong Kong, P. R. China

**Keywords:** Photochemistry, Organometallic chemistry

## Abstract

Photochromic materials have drawn growing attention because using light as a stimulus has been regarded as a convenient and environmental-friendly way to control properties of smart materials. While photoresponsive systems that are capable of showing multiple-state photochromism are attractive, the development of materials with such capabilities has remained a challenging task. Here we show that a benzo[*b*]phosphole thieno[3,2*‑b*]phosphole-containing alkynylgold(I) complex features multiple photoinduced color changes, in which the gold(I) metal center plays an important role in separating two photoactive units that leads to the suppression of intramolecular quenching processes of the excited states. More importantly, the exclusive photochemical reactivity of the thieno[3,2*‑b*]phosphole moiety of the gold(I) complex can be initiated upon photoirradiation of visible light. Stepwise photochromism of the gold(I) complex has been made possible, offering an effective strategy for the construction of multiple-state photochromic materials with multiple photocontrolled states to enhance the storage capacity of potential optical memory devices.

## Abstract (web summary)

Photoresponsive compounds have potential applications in various fields, including the development of smart materials and switches. Here the authors report a gold(I) complex that undergoes multiple photoinduced color changes upon excitation of light at specific wavelengths, offering an enhanced storage capacity towards optical memory devices.

## Introduction

Owing to the growing demands of smart materials that can alter their physical and chemical properties for potential applications, stimuli-responsive compounds with possible stimuli-controlled functions have drawn enormous attention over decades^1–4^. In particular, the photoresponsive system is amongst one of the most attractive and important candidates in stimuli-responsive systems^5–9^ because the stimulus of light has been regarded to be environmentally benign, convenient to access and orthogonal to electronic components of devices. Upon varying the wavelength and intensity, remote control using light could be accomplished in a qualitative and quantitative manner, respectively^5^. In addition, it is easier to deliver light as a stimulus to achieve remote control on the smart materials over time and space with high precision when compared to other stimuli^6^. The potential applications of photoresponsive materials, such as in optical memory storage^10^ and photoswitchable molecular devices^11^, have continuously provided the motivation for the rapid development of new photoresponsive materials with promising properties. Among the photoresponsive molecules, diarylethene has been recognized as one of the most popular molecules^12–23^ because of the excellent thermal stability of the photogenerated colored closed form that leads to high thermal irreversibility, robust photostability over multiple photoswitching cycles that offers its high fatigue resistance, distinctly different photophysical properties of the thermodynamically stable open and photogenerated closed forms, and relatively high photoswitching efficiency^12–17^.

**Fig. 1:**
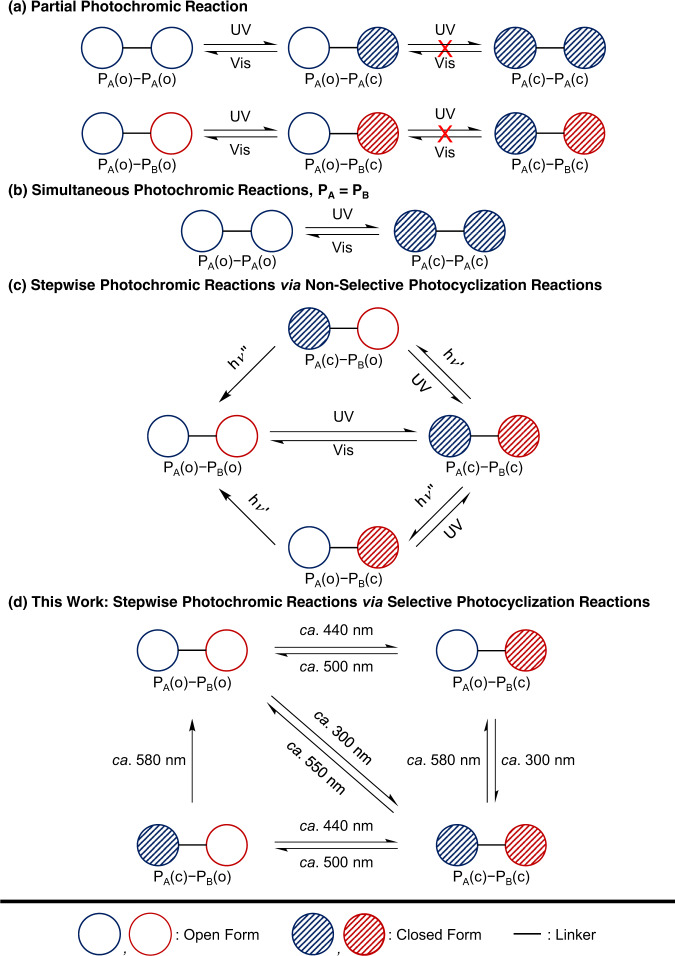
Different scenarios in photochromic properties of multiple diarylethene-containing systems. **a** Partial photochromic reaction: the system showing a single photochromic reaction due to the quenching process which prohibits the second photochromic reaction. **b** Simultaneous photochromic reactions: the system displaying multiple photochromic reactions at the same time to result in a single photoinduced color change. **c** Stepwise photochromic reactions via nonselective photocyclization reactions: the system showing simultaneous photocyclization reactions, followed by selective photocycloreversion reactions of individual photogenerated closed forms upon photoexcitation at specific wavelengths. **d** Stepwise photochromic reactions via selective photocyclization reaction: the system in this work featuring an exclusive photocyclization reaction of the visible-light-driven photoswitch, followed by a subsequent photocyclization reaction of the UV-driven photoswitch, and the selective photocycloreversion reactions of their closed forms by photoexcitation at different wavelengths. P_A_ and P_B_ denote photoswitches A and B, respectively, and (o) and (c) denote open and closed forms, respectively.

In general, photoresponsive molecules should ideally undergo reversible photochemical reactions upon excitation of light, providing a bistable system coded with ON and OFF states^12^. Furthermore, a photoresponsive system that is able to offer more than two states by photocontrol so as to enrich the diversity and the versatility and to enhance the storage capacity of optical memory devices, would be much more appealing than the conventional bistable photoresponsive systems^24–26^. However, the development of multiple-state photochromic systems with photocontrolled multistates has remained a major challenge, with only a limited number of compounds capable of successfully exhibiting multiple photoinduced color changes^24^. Since 2003, Irie and coworkers have reported the pioneering works of a dimer^27^ and a trimer^28^ of organic fused-diarylethenes with multiple photochromism that display up to four colors. A decade later, Chen and coworkers reported a few examples of Au(I)^29^, Pt(II)^30^, and Ru(II)^31^ complexes by the incorporation of different diarylethene-based ligands to undergo multiple photocyclization reactions with multistates in four colors. Except for the above-mentioned examples, most of the other systems with more than one diarylethene unit, have only shown a single photochromic reaction^32–38^ because of the presence of intramolecular energy transfer quenching of the photoactive open form excited states by the photogenerated closed form (Fig. 1a)^34^. Another challenge in the design of multiple-state photochromic systems is the need for orthogonality of the photochromic and photophysical properties of the diarylethene units^24^. It has commonly been observed that for a system consisting of more than one diarylethene unit, all the open forms could undergo photocyclization reactions simultaneously and similarly, the closed forms could undergo photocycloreversion reactions at the same time (Fig. 1b)^39–46^, resulting in a single photoinduced color change, unless the photocyclization and photocycloreversion reactions of different diarylethene units could be orthogonal and triggered selectively by photoexcitation at different wavelengths. Despite multiple photochromism demonstrated by the aforementioned examples, they are realized mainly by the simultaneous photocyclization reactions of all open form moieties nonselectively upon UV excitation (Fig. 1c)^27–31^, followed by subsequent photocycloreversion reactions of each closed form unit. Therefore, there is a strong need for the design of multiple photochromic systems that can allow selective and versatile control of a photocyclization reaction of the desired open form unit in an exclusive manner for potential applications of multiple-state photochromic materials.

Luminescent gold(I) complexes have been extensively explored and utilized for potential applications^47^ including chemosensors for the detection of various metal ions^48^, supramolecular self-assembly materials^49–55^, aurophilicity-induced stimuli-responsive materials^56^ and catalysis^57^. With the simple preparation methods, relatively good thermal- and photo-stabilities as well as unique linear geometry^58^, alkynylgold(I) complexes represent one of the most interesting members among the family of organogold(I) complexes. Recent work by us showed that alkynylgold(I) complexes could be used to construct multi-stimuli-responsive materials^59^, in which photochromic and mechanochromic moieties could be engineered into the same molecule with the two functions independent of each other via gold(I) complexation. It is believed that the gold(I) center can play an important role in isolating the different stimuli-responsive moieties through appropriate design^59^.

Considering the promising photochromic properties of phosphole-fused dithienylethene-based materials^59–62^, together with the rare examples of multiple-state photochromic materials to afford photocontrolled multistates^24^, we envision that the incorporation of two dithienylethene-based photoswitches of the UV-driven benzo[*b*]phosphole^60^ and the visible-light-driven thieno[3,2*‑b*]phosphole^61^ to gold(I) via metal complexation could realize a successful demonstration of multiple photochromism. More interestingly, the gold(I) metal center plays an important role in separating two photoactive units that lead to the suppression of intramolecular quenching processes of the excited states^63^. Furthermore, it is also anticipated that an exclusive photochemical reactivity could be switched on only on the visible-light-driven thieno[3,2*‑b*]phosphole rather than the UV-driven benzo[*b*]phosphole moieties upon photoexcitation of visible light, allowing an occurrence of a single photocyclization reaction of the selective photoswitchable unit (Fig. 1d). Besides, the individual photocycloreversion reaction of the closed forms of benzo[*b*]phosphole and thieno[3,2*‑b*]phosphole ligands would be initiated independently upon photoexcitation of visible light at different wavelengths. With the continuous interest in exploring various sophisticated photochromic systems^34–38,46,59–62,64–70^, in this work, we report the multiple-state photochromic benzo[*b*]phosphole thieno[3,2*‑b*]phosphole-containing alkynylgold(I) complex **1**, including its synthesis and characterization, together with the electrochemical, photophysical and multiple-state photochromic properties, in which reversible multistates can be photocontrolled qualitatively by an external stimulus of light with different wavelengths. More importantly, complex **1** has been found to show excellent thermal irreversibility even at high temperature, robust fatigue resistance at ambient condition, high conversion of the closed forms at the photostationary state (PSS) and good photoswitching efficiency, rendering it an important candidate of multiple-state photochromic organometallic systems with multiple states for the potential applications of optical memory devices with enhanced storage capacity in the foreseeable future.

## Results and discussion

### Synthesis and structural characterization

**Fig. 2:**
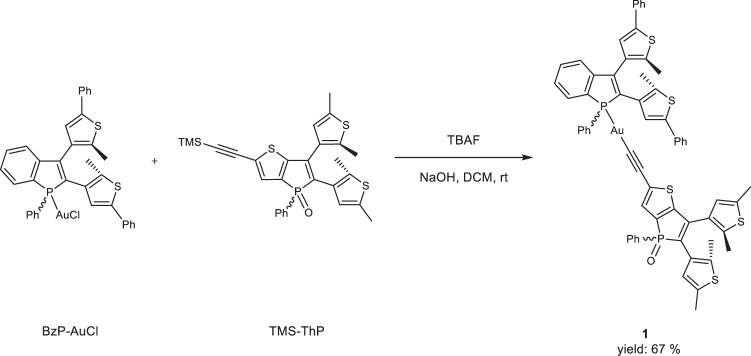
Synthetic route of the benzo[*b*]phosphole thieno[3,2*‑b*]phosphole-containing alkynylgold(I) complex **1**. TBAF: tetra-*n*-butylammonium fluoride, NaOH: sodium hydroxide, DCM: dichloromethane, rt: room temperature.

The multiple-state photochromic benzo[*b*]phosphole thieno[3,2*‑b*]phosphole-containing alkynylgold(I) complex **1** was synthesized by a modified literature procedure for the synthesis of benzo[*b*]phosphole alkynylgold(I) complexes (Fig. 2)^59^. Complex **1** has been readily prepared by mixing the benzo[*b*]phosphole chlorogold(I) precursor (BzP-AuCl), TMS-protected thieno[3,2*‑b*]phosphole-containing alkyne (TMS-ThP), tetra-*n*-butylammonium fluoride (TBAF) and sodium hydroxide in dichloromethane solution at room temperature overnight. Complex **1** was obtained after column chromatography and recrystallization as a yellow solid with good yield and is shown to be stable to air and moisture. Interestingly, complex **1** can be dissolved in a wide range of nonpolar to polar solvents such as hexane, benzene, dichloromethane, ethyl acetate, and methanol. Complex **1** was characterized by ^1^H and ^31^P{^1^H} NMR spectroscopy, HR-ESI-MS and elemental analysis. Owing to the presence of diastereomeric mixtures with the existence of a chiral center at the phosphorus atom and the helical structure rendered by the disposition of the two photoactive bis-thienyl rings, the benzo[*b*]phosphole moiety of complex **1** is found to feature two sets of ^1^H and ^31^P{^1^H} NMR signals (Supplementary Figs. 1 and 2) even at high temperature (70 °C). This has also been observed in the previously reported benzo[*b*]phosphole derivatives^60^. In contrast, the thieno[3,2*‑b*]phosphole moiety shows only one set of signals in the ^1^H and ^31^P{^1^H} NMR spectra at high temperature (70 °C)^61^, leading to the observation of two sets of ^31^P{^1^H} NMR signals (*δ* 46.94 and 47.08 ppm) for the benzo[*b*]phosphole moiety and a set of ^31^P{^1^H} NMR signal (*δ* 29.41 ppm) for the thieno[3,2*‑b*]phosphole moiety in complex **1**.

### Electrochemistry and photophysics

Complex **1** in dichloromethane (0.1 M ^*n*^Bu_4_NPF_6_) is found to display an irreversible oxidation wave at +1.17 V and two irreversible reduction waves at −1.66 V and −1.85 V vs. saturated calomel electrode (SCE) (Supplementary Table 1). With a potential value similar to that of the oxidation of the thiophene-containing alkyne^37^, the irreversible oxidation wave of complex **1** is attributed to the gold(I)-perturbed thiophene-containing alkynyl-based oxidation (Supplementary Fig. 3). With reference to the thieno[3,2*‑b*]phosphole oxides^61^ and the benzo[*b*]phosphole gold(I) complexes^59^, the first and second irreversible reduction waves of complex **1** are assigned as the gold(I)-perturbed thieno[3,2*‑b*]phosphole oxide-based and benzo[*b*]phosphole-based reduction (Supplementary Fig. 4), respectively. Complex **1** in benzene solution shows strong absorptions in the UV region at about 290–340 nm (Supplementary Fig. 5 and Supplementary Table 2), which are tentatively assigned as a mixture of gold(I)-perturbed intraligand [π → π*] transitions of the dithienyl-substituted benzo[*b*]phosphole and thieno[3,2*‑b*]phosphole-containing alkynyl ligands, since BzP-AuCl and TMS-ThP are also found to absorb strongly in this UV region (Supplementary Fig. 5). Moreover, the lowest-energy absorption band in the visible region at 360–490 nm of complex **1** is assigned to the gold(I)-perturbed intraligand [π → π*] transitions of the dithienyl-substituted thieno[3,2*‑b*]phosphole-containing alkynyl ligand because only the visible-light-driven photoswitchable alkynyl ligand absorbs in the visible region (Supplementary Fig. 5). Complex **1** displays green fluorescence with an emission maximum at *ca.* 550 nm upon photoexcitation at about 360–450 nm (Supplementary Fig. 6 and Supplementary Table 2). Given the insensitivity to oxygen, relatively small Stokes shift (Supplementary Fig. 7), emission lifetime shorter than a submicrosecond, together with the similarity of the emission spectra of the alkynylgold(I) complex with TMS-ThP (Supplementary Fig. 6), the fluorescence of complex **1** is originated from the singlet gold(I)-perturbed [π−π*] thieno[3,2*‑b*]phosphole-containing alkynyl ligand-centered excited state with intraligand charge transfer character. Complex **1** is found to exhibit much lower luminescence quantum yield (*ϕ*_lum_ = 3.3%) than the reported phosphole-based compounds^71,72^, likely due to the existence of the photochemically active dithienylethene-based benzo[*b*]phosphole and thieno[3,2*-b*]phosphole units, providing competitive pathways for photocyclization reactions other than luminescence in the excited state.

### Multiple-state photochromism

**Fig. 3:**
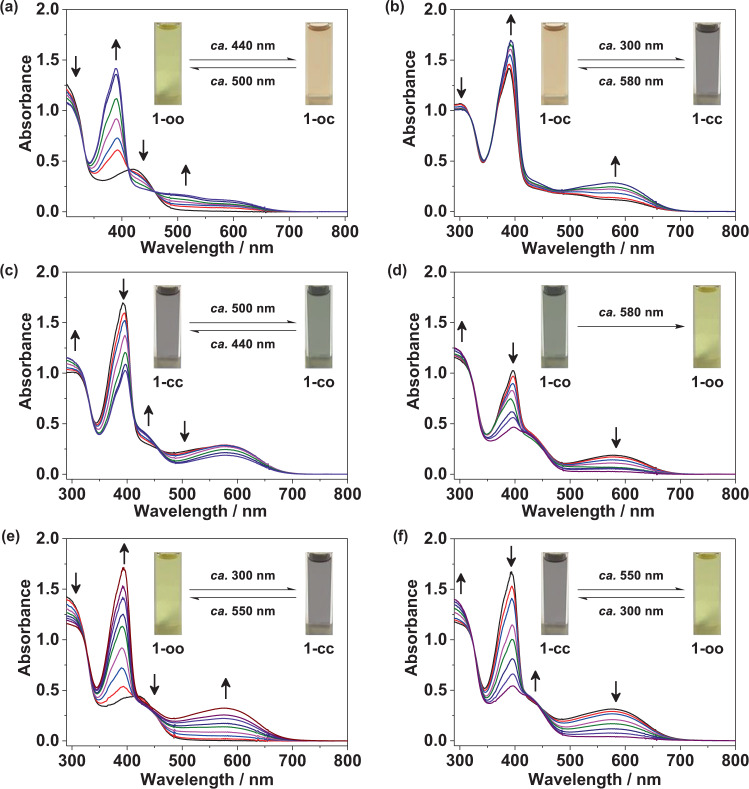
UV–Vis spectral traces of the stepwise photochromic reactions of complex **1**. **a** Photocyclization reaction of **1-oo** to form **1-oc** upon excitation of visible light at *ca*. 440 nm. **b** Photocyclization reaction of **1-oc** to form **1-cc** upon UV excitation at *ca.* 300 nm. **c** Photocycloreversion reaction of **1-cc** to form **1-co** upon excitation of visible light at *ca*. 500 nm. **d** Photocycloreversion reaction of **1-co** to form **1-oo** upon excitation of visible light at *ca*. 580 nm. **e** Simultaneous photocyclization reactions of **1-oo** to form **1-cc** upon UV excitation. **f** Simultaneous photocycloreversion reactions of **1-cc** to form **1-oo** upon excitation of visible light at *ca.* 550 nm.

**Fig. 4:**
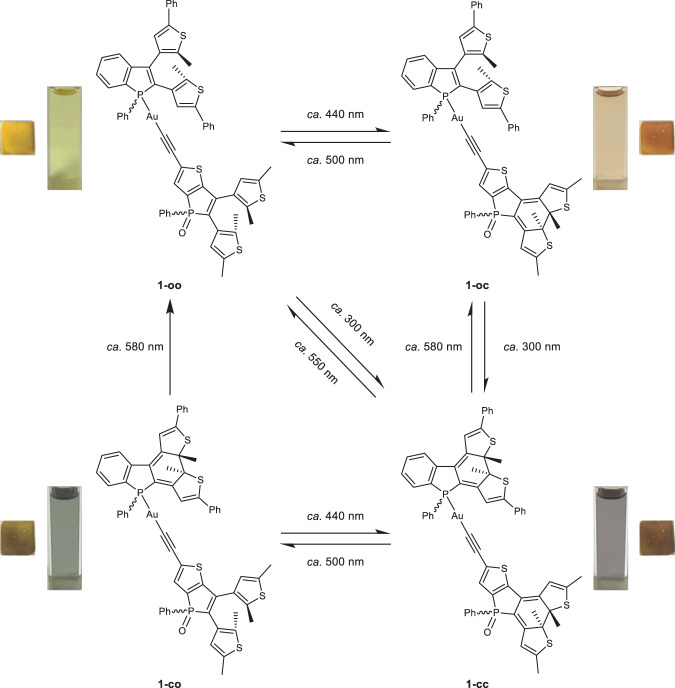
Stepwise photochromic reactions of complex **1** upon photoexcitation of light at selective wavelengths. Insets show the multiple photoinduced color changes of complex **1** in benzene solution and PMMA thin film.

Upon photoirradiation at *ca.* 440 nm, the benzene solution of complex **1** features photoinduced color changes from yellow to orange, with the emergence of a new low-energy absorption band with a maximum at *ca*. 500 nm (Fig. 3a), which is attributed to the photochemical cyclization of the open form of thieno[3,2*‑b*]phosphole moiety (**1-oo** → **1-oc**). Subsequent UV excitation at *ca*. 300 nm led to photocyclization of the open form of the benzo[*b*]phosphole moiety (**1-oc** → **1-cc**), showing color changes from orange to purplish-gray with the appearance of a new low-energy absorption band at *ca*. 580 nm (Fig. 3b). Upon subsequent photoirradiation of visible light at *ca*. 500 nm, photocycloreversion reaction of the closed form of thieno[3,2*‑b*]phosphole moiety could occur predominantly (**1-cc** → **1-co**), in which color changes from purplish-gray to greenish-gray, with a drop of the low-energy absorption band at *ca*. 500 nm, are observed (Fig. 3c). Eventually, a recovery of the yellow solution from the greenish-gray solution has been realized upon photoexcitation at *ca*. 580 nm, with a drastic reduction of the lowest-energy absorption band at *ca*. 580 nm (Fig. 3d), indicative of the photochemical cycloreversion of the closed form of benzo[*b*]phosphole moiety (**1-co** → **1-oo**). On the other hand, both the open forms of benzo[*b*]phosphole and thieno[3,2*‑b*]phosphole ligands could undergo simultaneous photocyclization reactions upon UV excitation (**1-oo** → **1-cc**), with the color of the solution changing from yellow to purplish-gray, concomitant with the growth of new low-energy absorption bands at *ca*. 500–700 nm (Fig. 3e). However, shining visible light at *ca*. 550 nm on the closed forms could lead to complete photocycloreversion reactions (**1-cc** → **1-oo**), featuring photoinduced color changes from purplish-gray to yellow in solution with a dramatic drop in the absorption bands of the closed forms (Fig. 3f). In contrast, photocycloreversion reaction of the closed form of the benzo[*b*]phosphole moiety (**1-cc** → **1-oc**) is triggered predominantly upon photoexcitation at *ca*. 580 nm, in which the UV–vis absorption spectral changes are shown in Supplementary Fig. 8. There are no obvious changes at *ca*. 410–460 nm, where the thieno[3,2‑*b*]phosphole moiety would feature observable changes if there were photochromic reactions (Fig. 3a, c), indicating no significant photocycloreversion reaction of the closed form of the thieno[3,2‑*b*]phosphole moiety could occur upon photoexcitation at *ca*. 580 nm. Clear isosbestic points are observed in all the UV–vis absorption spectral changes of each photochemical process, suggesting clean conversions between the open forms and closed forms of both the benzo[*b*]phosphole and thieno[3,2*‑b*]phosphole units (**1-oo**, **1-oc**, **1-cc**, and **1-co**). Interestingly, the conversion from **1-oc** to **1-cc** involves photocyclization reaction of the benzo[*b*]phosphole moiety, showing smaller changes at 390 nm in UV–vis absorption spectral traces (Fig. 3b); while the changes at 390 nm are found to be much larger during photocycloreversion reaction of the thieno[3,2‑*b*]phosphole moiety (Fig. 3c), leading to conversion from **1-cc** to **1-co**. Since the molecular structure of complex **1** consists of two photochemically active ligands of dithienylethene-based benzo[*b*]phosphole and thieno[3,2*‑b*]phosphole units linked together via gold(I) complexation, it is anticipated that complex **1** is capable of performing multiple photochromism upon photoirradiation of selective wavelengths (Fig. 4). As the benzo[*b*]phosphole and thieno[3,2*‑b*]phosphole units are responsible for the respective UV and visible-light-triggered photocyclization, it is envisioned that visible light excitation of complex **1** could only bring about photocyclization of the photochromic thieno[3,2*‑b*]phosphole ligand. Apart from the UV–vis absorption spectroscopy, ^1^H and ^31^P{^1^H} NMR spectroscopy have also been employed to probe the processes of the photocyclization and photocycloreversion reactions (Supplementary Figs. 9 and 10). The ^1^H and ^31^P{^1^H} NMR spectral changes during stepwise photochromism have clearly shown the existence of photogenerated closed forms of the benzo[*b*]phosphole and thieno[3,2*‑b*]phosphole moieties without any significant side-product formation. More importantly, the exclusive formation of **1-oc** by specific photocyclization of the open form of the thieno[3,2*‑b*]phosphole moiety could be achieved by photoirradiation with visible light at *ca*. 440 nm on **1-oo**, which has been supported by the growth of new ^31^P{^1^H} NMR signals at *δ* 17.02 and 18.45 ppm (Supplementary Fig. 10). Furthermore, the generation of **1-co** could also be realized by photoexcitation at *ca*. 500 nm of **1-cc** via the predominant photocycloreversion reaction of the closed form of the thieno[3,2*‑b*]phosphole moiety, displaying the appearance of new ^31^P{^1^H} NMR signals at *δ* 28.50 and 29.61 ppm (Supplementary Fig. 10). For the lowest-energy absorption maxima of the closed forms, the phenyl-substituted 1,8-thia-*as*-indacene of benzo[*b*]phosphole moiety (*λ*_**1-co**_ = 580 nm) has been found to exhibit a bathochromic shift relative to the methyl-substituted 1,8-thia-*as*-indacene of the thieno[3,2*‑b*]phosphole unit (*λ*_**1-oc**_ = 500 nm) (Supplementary Fig. 11 and Supplementary Table 2). The UV − vis absorption spectral changes of BzP-AuCl and TMS-ThP upon photocyclization reaction have been performed for a comparison of the absorption properties of the closed forms (Supplementary Figs. 12 and 13), in which the formation of the closed forms has also been monitored by the ^31^P{^1^H} NMR spectroscopy (Supplementary Fig. 14). Interestingly, the ^31^P{^1^H} NMR signals of both the open form and the closed form of TMS-ThP (open: *δ* 26.09; closed: *δ* 16.39 and 17.89) are shown to be similar to those of the thieno[3,2‑*b*]phosphole moiety of **1** (open: *δ* 26.74; closed: *δ* 17.02 and 18.45). However, the ^31^P{^1^H} NMR signals of the open form and the closed form of BzP-AuCl (open: *δ* 29.18 and 30.56; closed: *δ* 16.22) are found to be more upfield shifted than those of the benzo[*b*]phosphole moiety of **1** (open: *δ* 47.38 and 47.44; closed: *δ* 28.50 and 29.61), which had also been observed in the previously reported benzo[*b*]phosphole alkynylgold(I) complexes^59^. In addition, complex **1** displays a photoinduced decline in the intensity of the green emission (*λ*_**em**_ = 550 nm) with an 80% decrease during the photocyclization reactions with UV excitation (Supplementary Fig. 15). Similarly, BzP-AuCl and TMS-ThP also show emission spectral changes with a drop of the emission intensity upon photocyclization reactions (Supplementary Figs. 16 and 17).

Complex **1** is shown to exhibit promising photochromic properties, including excellent thermal irreversibility, robust fatigue resistance under ambient conditions, high conversion of the photogenerated closed form from the open form at the PSS, and good photochemical cyclization and cycloreversion quantum yields to offer good photoswitching efficiency. Intriguingly, both the closed forms of benzo[*b*]phosphole and thieno[3,2*‑b*]phosphole moieties of complex **1** feature negligible thermal backward ring-opening reactions (<0.1%) even at a higher temperature of 50 °C over 4000 min (Supplementary Figs. 18 and 19). Moreover, the closed forms of complex **1** also display over ten photochromic cycles without significant photodegradation (<2.2%) under ambient conditions, with no obvious loss of photochemical activity at each cycle, suggesting remarkable photoreversibility from the closed forms to the open forms (Supplementary Figs. 20 and 21). The conversion from the open form to the photogenerated closed forms of the benzo[*b*]phosphole and thieno[3,2*‑b*]phosphole moieties in complex **1** is found to be 90% and 87% (Supplementary Table 3), respectively. The photocyclization quantum yields of the open forms of benzo[*b*]phosphole and thieno[3,2*‑b*]phosphole moieties of complex **1** are found to be 29% and 64%, respectively, whereas the photocycloreversion quantum yields are determined to be 1.1% and 8.2% for the closed forms of benzo[*b*]phosphole and thieno[3,2*‑b*]phosphole moieties (Supplementary Table 3), respectively. Considering the potential application of photochromic materials for optical memory devices with enhanced storage density, solid-state photochromism of complex **1** on thin film has been explored to provide multiple photocontrolled state of different colors. Upon photoirradiation at selective wavelengths, four isomers (**1-oo**, **1-oc**, **1-cc**, **1-co**) of the alkynylgold(I) complex **1** on the PMMA thin film with yellow, orange, brown and bronze olive in colors can be successfully obtained (Fig. 4).

### Computational study

**Fig. 5:**
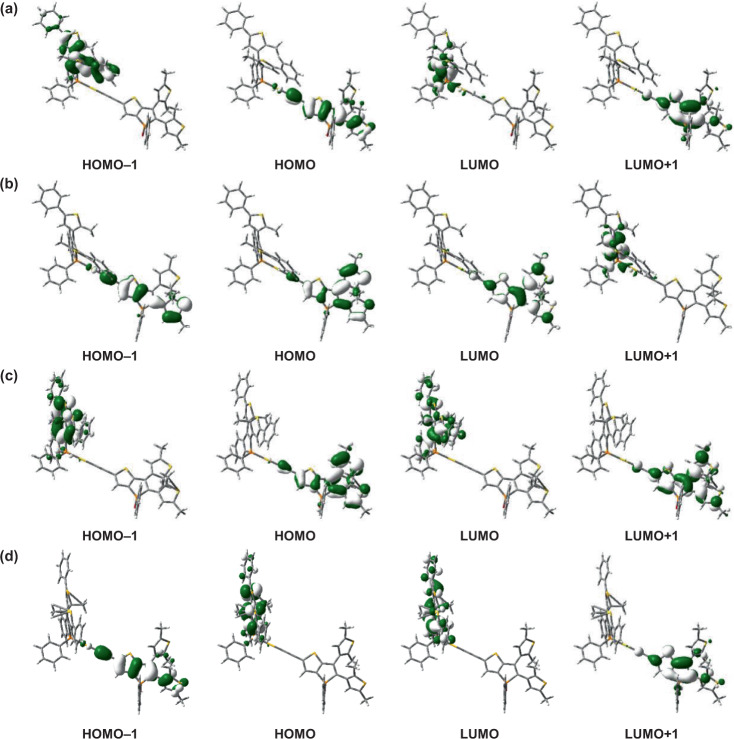
Spatial plots (isovalue = 0.03) of selected molecular orbitals of complex 1 at the optimized ground-state geometry. **a** Selected molecular orbitals of **1-oo** that contains both the open forms of benzo[*b*]phosphole and thieno[3,2-*b*]phosphole moieties. **b** Selected molecular orbitals of **1-oc** that contains the open form of benzo[*b*]phosphole moiety and the closed form of thieno[3,2-*b*]phosphole moiety. **c** Selected molecular orbitals of **1-cc** that contains both the closed forms of benzo[*b*]phosphole and thieno[3,2-*b*]phosphole moieties. **d** Selected molecular orbitals of **1-co** that contains the closed form of benzo[*b*]phosphole moiety and the open form of thieno[3,2-*b*]phosphole moiety.

**Fig. 6:**
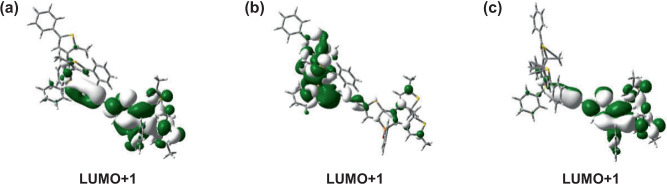
Spatial plots of the virtual orbitals (with a smaller isovalue of 0.01) allowing photocyclization reaction in complex 1. **a** The LUMO+1 of **1-oo** that contains both the open forms of benzo[*b*]phosphole and thieno[3,2-*b*]phosphole moieties. **b** The LUMO+1 of **1-oc** that contains the open form of benzo[*b*]phosphole moiety and the closed form of thieno[3,2-*b*]phosphole moiety. **c** The LUMO+1 of **1-co** that contains the closed form of benzo[*b*]phosphole moiety and the open form of thieno[3,2-*b*]phosphole moiety.

To gain more insight into the multiple-state photochromic properties of complex **1**, density functional theory (DFT) and time-dependent density functional theory (TDDFT) calculations have been performed on **1-oo**, **1-oc**, **1-cc**, and **1-co** (Figs. 5, 6 and Supplementary Fig. 22). The lowest-energy absorption band of **1-oo**, computed at *ca*. 441 nm (Supplementary Fig. 23 and Supplementary Table 4), is attributed to the HOMO → LUMO+1 excitation, where the HOMO and the LUMO+1 are the π and π* orbitals, respectively, predominantly localized on the dithienylethene-containing thieno[3,2-*b*]phosphole alkynyl ligand (Fig. 5a). Therefore, the lowest-energy absorption band can be assigned as IL [π → π*] transition of the open form of the thieno[3,2-*b*]phosphole alkynyl ligand. The high-energy absorption band computed at *ca*. 302–317 nm is predominantly contributed by the IL [π → π*] transition of the open form of benzo[*b*]phosphole moiety. Upon photocyclization of the open form of the thieno[3,2*‑b*]phosphole moiety in **1-oo**, **1-oc** is formed and exhibits a low-energy absorption band at *ca*. 514 nm (Supplementary Fig. 24 and Supplementary Table 5). This band is contributed by the HOMO → LUMO excitation, where the HOMO and LUMO are the π and π* orbitals, respectively, localized on the methyl-substituted 1,8-thia-*as*-indacene condensed ring (Fig. 5b), and thus it can be assigned as IL [π → π*] transition of the closed form of the thieno[3,2*‑b*]phosphole moiety. Upon further photocyclization of the open form of the benzo[*b*]phosphole moiety in **1-oc**, **1-cc** is formed and shows a low-energy absorption band at *ca*. 608 nm (Supplementary Fig. 25 and Supplementary Table 6), which is attributed to the HOMO−1→LUMO excitation. The HOMO−1 and LUMO of **1-cc** are the π and π* orbitals, respectively, on the phenyl-substituted 1,8-thia-*as*-indacene condensed ring moiety (Fig. 5c), and therefore, it can be assigned as IL [π → π*] transition of the closed form of benzo[*b*]phosphole. Moreover, upon photocycloreversion of the closed form of the thieno[3,2*‑b*]phosphole moiety in **1-cc**, the lowest-energy absorption band at *ca*. 608 nm remains, but the singlet-singlet transition computed at *ca*. 514 nm in **1-cc** (S_0_ → S_3_) is found to disappear in **1-co** (Supplementary Fig. 26 and Supplementary Table 7), which is consistent with a drop of the low-energy absorption band at *ca*. 500 nm observed in the photochromic study. Moreover, **1-co** displays a low-energy absorption band at *ca*. 608 nm that is originated from the HOMO → LUMO excitation. As the HOMO and LUMO are the π and π* orbitals localized on the phenyl-substituted 1,8-thia-*as*-indacene condensed ring (Fig. 5d), IL [π → π*] transition of the closed form of benzo[*b*]phosphole moiety is responsible for the lowest-energy absorption band of **1-co**. Finally, the photocycloreversion of the closed form of the benzo[*b*]phosphole moiety in **1-co** leads to the formation of **1-oo** with a decline of the lowest-energy absorption band at *ca*. 580 nm observed in the experiment. The experimental UV–vis spectra of the four isomers, namely **1-oo**, **1-oc**, **1-cc**, and **1-co**, are in good agreement with the simulated UV–vis spectra (Supplementary Figs. 23–26).

To assess the possibility of photocyclization reactions in complex **1**, the distances between the photoreactive carbon atoms, the relative Gibbs free energies of the closed forms (**1-oc**, **1-cc**, and **1-co**) with respect to the open form (**1-oo**), and the virtual orbitals responsible for photocyclizations of the open forms of benzo[*b*]phosphole and thieno[3,2*-b*]phosphole moiety have been taken into account for multiple-state photochromic behaviors of complex **1**. The distances between the two photoreactive carbon atoms of the open forms of the benzo[*b*]phosphole and thieno[3,2*-b*]phosphole moieties in **1-oc** and **1-co**, respectively, are 3.62 and 3.56 Å (Supplementary Table 8), which are almost identical to those in **1-oo**. The Gibbs free energies of **1-oc**, **1-cc**, and **1-co**, with respect to **1-oo**, are 0.67, 11.90, and 10.71 kcal mol^−1^, respectively (Supplementary Table 8), revealing that **1-oc** is more stable than **1-co** and **1-cc**. The low-energy absorption band at 441 nm of **1-oo** is contributed by the HOMO → LUMO+1 excitation, where the electron density of the LUMO+1 is localized on the open form of the thieno[3,2*-b*]phosphole moiety. The signs of the lobes on the *p* orbitals of the reactive carbon atoms are preorganized for the formation of a carbon–carbon σ bond via a conrotatory fashion (Fig. 6a), providing a geometric requirement of the molecular orbital for **1-oo** to undergo photocyclization to give **1-oc** upon photoirradiation at *ca*. 440 nm. Regarding **1-oc**, the transitions computed at 514 nm (HOMO → LUMO) and 395 nm (HOMO−1→LUMO and HOMO → LUMO + 2) would not be able to bring about a ring closure, as there is no electron density localized on the reactive carbon atoms of the open form of the benzo[*b*]phosphole moiety in the LUMO as well as the lack of possible bond formation for a carbon–carbon σ bond in a conrotatory fashion in the LUMO + 2. The photocyclization at the open form of the benzo[*b*]phosphole moiety is instead contributed by the S_0_ → S_15_ transition at 318 nm, which corresponds to the HOMO − 5→LUMO+1 excitation. This is because, in the LUMO+1, the signs of the lobes on the *p* orbitals of the reactive carbon atoms are preorganized to enable the formation of the carbon–carbon σ bond in a conrotatory fashion (Fig. 6b). For **1-co**, the S_0_ → S_1_ transition computed at 608 nm (HOMO → LUMO) would not result in the ring closure of the open form of the thieno[3,2*-b*]phosphole moiety because there is no electron density localized on the reactive carbon atoms of the open form in the LUMO. However, the ring closure of the thieno[3,2*-b*]phosphole moiety is induced by the S_0_ → S_3_ transition at 443 nm (HOMO−1→LUMO+1), due to the signs of the lobes on the *p* orbitals of the reactive carbon atoms in LUMO+1 are preorganized to allow the formation of the carbon–carbon σ bond in a conrotatory fashion (Fig. 6c). From the perspectives of molecular geometry, energetic features and virtual orbitals of complex **1**, the realization of multiple-state photochromic behaviors has been strongly supported by the computational study. The Cartesian coordinates of the optimized S_0_ geometries of **1-oo**, **1-oc**, **1-cc**, and **1-co** are given in Supplementary Tables 9–12, respectively.

Furthermore, the spin-orbital coupling (SOC) constants of **1-oo**, **1-oc**, and **1-co** have been determined using Amsterdam Density Functional (ADF) program, in order to gain more insights into the mechanism of the multiple-state photochromic reaction in **1**. These involve the estimation of the rate of intersystem crossing (ISC) in **1-oo**, **1-oc**, and **1-co**, which would be revealed by the SOC constants between the singlet excited states (S_n_) and the triplet excited states (T_m_) that are energetically close to S_n_ (Supplementary Table 13). The SOC constants are shown to be very small in **1-oo** (<S_1_ | *Ĥ*_SO_ | T_2_>_**1-oo**_ = 3.73 cm^−1^; <S_1_ | *Ĥ*_SO_ | T_3_>_**1-oo**_ = 2.24 cm^−1^), indicating that S_1_ → T_2_ ISC and S_1_ → T_3_ ISC processes in **1-oo** are inefficient. With reference to the reported systems having <S_n_ | *Ĥ*_SO_ | T_m_> values from 76.9 to 129 cm^−1^ to allow triplet excited state photocyclization^63^, much smaller values of SOC constants are found in **1-oc** and **1-co** (<S_4_ | *Ĥ*_SO_ | T_8_>_**1-oc**_ = 1.16 cm^−1^; S_4_ | *Ĥ*_SO_ | T_9_>_**1-oc**_ = 30.28 cm^−1^; <S_3_ | *Ĥ*_SO_ | T_4_>_**1-co**_ = 20.94 cm^−1^; <S_3_ | *Ĥ*_SO_ | T_5_>_**1-co**_ = 2.88 cm^−1^), suggesting the inefficient ISC processes in **1-oc** and **1-co**. Moreover, the energy difference between S_4_ and T_9_ in **1-oc** and that between S_3_ and T_4_ in **1-co** are estimated to be 0.18 and 0.30 eV, respectively, which are not negligibly small. Since the energy difference between S_n_ and T_m_ has an exponential effect on the rate of the ISC, together with the relatively low value of SOC constants in **1-oo**, **1-oc**, and **1-co**, inefficient ISC between the S_n_ and T_m_ states is anticipated. Therefore, the first photocyclization in **1-oo** and the second photocyclizations in **1-oc** and **1-co** would be mainly attributed to their singlet excited states, though the involvement of the triplet state via ISC to some “dark” triplet states cannot be completely ruled out. However, given the small SOC constants and the non-negligible energy differences between the S_n_ and T_m_ states, we believe this is less likely. Inefficient ISC in gold(I) systems is not unprecedented^73^. Given the inefficient ISC process associated with the relatively small SOC constants in **1-oo**, **1-oc**, and **1-co**, it is anticipated that the photocyclization of the open forms of benzo[*b*]phosphole and thieno[3,2‑*b*]phosphole moieties would occur within picosecond timescale via the singlet excited state, similar to that found for the photocyclization of the organic counterparts^74^.

In this work, a newly designed benzo[*b*]phosphole thieno[3,2*‑b*]phosphole-containing alkynylgold(I) complex has been successfully synthesized to display multiple photoinduced color changes upon selective photoexcitation of light at specific wavelengths. With the rare examples of multiple-state photochromic organometallic complexes, the present work has not only successfully illustrated that the multiple photoinduced color changes can be accomplished by the incorporation of UV-driven and visible-light-driven photoswitches with promising photochromic behaviors via gold(I) complexation but also has demonstrated that the metal center of gold(I) has played an important role in assisting the photochemical reactivity of the remaining open form unit instead of suffering from the quenching process. It is envisaged that the molecular design of this new benzo[*b*]phosphole thieno[3,2*‑b*]phosphole-containing alkynylgold(I) complex that features multiple-state photochromic properties will provide an in-depth insight into the construction of photochromic organometallic complexes with photocontrolled multiple states using an easy preparation method, boosting the storage capacity of optical memory devices for potential applications in the foreseeable future.

## Methods

Materials, spectroscopic measurements and instrumentation, and computational details are available in the Supplementary Information.

## Supplementary information

Supplementary information

## Data Availability

The data that support the plots within this paper and other findings of this study are available from the manuscript, its Supplementary Information, or from the corresponding author upon reasonable request. The electrochemical, photophysical, photochromic, and computational data generated in this study are provided in the Supplementary Information.
