# Supplementary material for: Photocontrolled multiple-state photochromic benzo[b]phosphole thieno[3,2-b]phosphole-containing alkynylgold(I) complex via selective light irradiation
Source: Nat Commun. 2022 Jan 10;13:33. doi: 10.1038/s41467-021-27711-9 (PMC8748877; doi:10.1038/s41467-021-27711-9)
Supplement: Supplementary file 1 — Supplementary information [file 41467_2021_27711_MOESM1_ESM.pdf]

Supplementary Information for

Photocontrolled Multiple-State Photochromic Benzo[*b*]phosphole Thieno[3,2-*b*]phosphole-Containing Alkynylgold(I) Complex *via* Selective Light Irradiation

Nathan Man-Wai Wu, Maggie Ng, Vivian Wing-Wah Yam*

Institute of Molecular Functional Materials, State Key Laboratory of Synthetic Chemistry and Department of Chemistry, The University of Hong Kong, Pokfulam Road, Hong Kong (China)

E-mail: wwyam@hku.hk; Fax: (852) 28571586; Tel: (852) 28592153

Experimental Section

Materials and Reagents

The photochromic benzo[*b*]phosphole chlorogold(I) precursor (BzP-AuCl)[1] and TMS-protected thieno[3,2-*b*]phosphole-containing alkynyl ligand (TMS-ThP)[2] were synthesized according to the literature procedures. If not specified, all solvents and reagents (analytical grade) were used without further purification and all reactions were performed under inert and anhydrous conditions using standard Schlenk techniques.

Physical Measurements and Instrumentation

The ^{1}H NMR spectra were recorded on a Bruker AVANCE 500 (500 MHz) Fourier transform NMR spectrometer and the $^{31}P\{^{1}H\}$ NMR spectra were recorded on a Bruker AVANCE 500 (203 MHz) Fourier transform NMR spectrometer. Tetramethylsilane (Me_4Si) and 85% phosphoric acid (H_3PO_4) were used as standard for the determination of the chemical shifts (δ, ppm) in the ^{1}H and $^{31}P\{^{1}H\}$ NMR spectroscopy, respectively. High-resolution electrospray ionization (HR-ESI) mass spectra were recorded on a Bruker maXis II High Resolution Liquid Chromatography Quadrupole-Time of Flight (LC-QTOF). A Carlo Erba 1106 elemental analyzer was employed for the determination of the elemental analyses of the new complexes (Institute of Chemistry, Chinese Academy of Sciences, Beijing). A CH Instrument, Inc. model CHI620 electrochemical analyzer was used for the cyclic voltammetric measurements. The electrochemical property of complex **1** was studied in deaerated dichloromethane solution in the presence of 0.1 M $^{n}Bu_4NPF_6$. A glassy carbon (CH Instrument) electrode and a Ag/$AgNO_3$ (0.1 M in acetonitrile) electrode were used as the working electrode and reference electrode, respectively. The counter electrode was a platinum wire that was separated from the glassy carbon (CH Instrument) electrode by a sintered-glass frit in the electrolytic cell. The internal reference was the ferrocenium/ferrocene couple ($FeCp^{2+/0}$).[3] A Varian Cary 50 UV–vis spectrophotometer was used for UV–vis absorption measurements. If not specified, solutions of complex **1** for photophysical and photochromic measurements were degassed with no less than four freeze–pump–thaw cycles on a high-vacuum line prior to measurements. Steady-state emission spectra of complex **1** were recorded on an Edinburgh Instrument FS5 spectrofluorometer. A conventional laser system was used for the excited-state lifetime measurements. The excitation source used was a 355-nm output (third harmonic, 8 ns) of a Spectra-Physics Quanta-Ray Q-switched GCR-150 pulsed Nd-YAG laser (10 Hz). Luminescence decay traces at a selected wavelength were detected by a Hamamatsu R928 photomultiplier tube and the voltage signal was recorded on a Tektronix Model TDS-620A digital oscilloscope (500 MHz, 2 GS/s). Optical dilute method was used for the determination of relative luminescence quantum yields using an aqueous solution of quinine sulfate in 0.5 M sulfuric acid (ϕ_{lum} = 0.546, λ_{ex} = 365 nm) as the standard at 298 K.[4] The kinetics experiments for the thermal backward reaction of the closed forms of complex **1** (for both benzo[*b*]phosphole and thieno[3,2-*b*]phosphole moieties) at various temperatures were carried out on a Varian Cary 50

UV–vis spectrophotometer with a single cell Peltier thermostat. Ferrioxalate actinometry was used for the determination of photochemical quantum yields.[5] The monochromatic light was generated by passing the light source from a 300 W Oriel Corporation Model 60011 Xe (ozone-free) lamp through an Applied Photophysics F 3.4 monochromator. The intensity of incident light at different wavelengths was obtained from the average values measured just before and after each photolysis experiment using ferrioxalate actinometry.[5] In the determination of photochromic quantum yields of complex **1** (for both benzo[*b*]phosphole and thieno[3,2-*b*]phosphole moieties), the sample solutions with absorbance slightly greater than 2.0 at the excitation wavelength were prepared and were subjected to photoirradiation. The photocyclization and photocycloreversion quantum yields were measured at a small percentage of conversion by observing the initial rate of absorbance changes ($\Delta A/\Delta t$) at the low-energy absorption maxima of the closed form.[5] The percentage conversions of the closed forms at their photostationary states (PSS) were quantified using a combination of ^{1}H and $^{31}P\{^{1}H\}$ NMR as well as UV–vis spectroscopic studies of irradiated sample with known concentration.

Computational Details

All calculations were performed with the Gaussian 09 suite of programs.[6] The ground-state (S_0) geometries of complexes **1-oo**, **1-oc**, **1-cc** and **1-co** were fully optimized in benzene by density functional theory (DFT) with the PBE0 hybrid functional,[7–9] in conjunction with the conductor-like polarizable continuum model (CPCM) using benzene as the solvent.[10,11] Vibrational frequencies were then calculated to verify that each was a minimum on the potential energy surface (PES), though a small imaginary frequency was found in the ground state of **1-co** (1i cm^{-1}). Based on the optimized S_0 geometries, time-dependent density functional theory (TDDFT) calculations were performed at the same level associated with CPCM, for the computation of the singlet-singlet transitions in the electronic absorption spectra of the four complexes. The Stuttgart effective core potentials (ECPs) and the associated basis set were employed to describe the Au[12] atom with f-type polarization functions ($\zeta = 1.050$),[13] whereas the 6-31G(d,p) basis set[14–16] was applied for all other atoms. All the DFT and TDDFT calculations were performed with a pruned (99,590) grid for numerical integration. The spin-orbital coupling (SOC) constants of **1-oo**, **1-oc** and **1-co** were computed by the Amsterdam Density Functional (ADF) program.[17,18]

Computational Study

The energy difference between the open form and the closed form in the thieno[3,2-*b*]phosphole moiety is found to be 0.67−1.21 kcal mol^{-1}, whereas that of the benzo[*b*]phosphole moiety is estimated to be 10.71−11.27 kcal mol^{-1} (Table S8). Interestingly, the thieno[3,2-*b*]phosphole moiety shows larger photocyclization and photocycloreversion quantum yields than that of the benzo[*b*]phosphole moiety (Table S3). The rather small Gibbs energy difference between **1-oo** and **1-oc** is unlikely to be a result of the small imaginary frequency found in **1-oo** because without thermal

correction, **1-oc** is still 0.17 kcal mol^{-1} less stable than **1-oo**. Moreover, geometry optimizations on the open and the closed forms of TMS-ThP have been performed. It was found that the closed form is only 3.6 kcal mol^{-1} higher than the open form, which can be considered as a small energy difference, and this is consistent with the small energy difference of 1.2 kcal mol^{-1} between **1-co** and **1-cc**. Besides, other conformers by performing geometry optimizations upon rotation around the central triple bond have also been tested. The result shows that there is no significant difference in the electronic energies of the optimized structures, as the energy differences are less than 0.1 kcal mol^{-1}. In addition, the orthogonality of the two DTEs has been investigated by observing the interplanar angle between the benzo[*b*]phosphole and the thieno[3,2-*b*]phosphole moieties. The interplanar angles in **1-oo**, **1-oc** and **1-co** are 84.8°, 81.5° and 74.7° respectively, indicating that the two DTEs are nearly perpendicular. Based on the experimental results, no significant excited-state energy transfer (EET) from the excited open form to the closed form moieties has been observed in **1-oc** and **1-co**. This has led to its ability to feature the second photocyclization reaction.

Synthesis and Characterization

The photochromic benzo[*b*]phosphole chlorogold(I) precursor (BzP-AuCl) was prepared using a modified literature procedure as described.[19] A mixture of chloro(tetrahydrothiophene)gold(I) (75 mg, 0.23 mmol) and 2,3-bis(2-methyl-5-phenylthiophen-3-yl)-1-phenylbenzo[*b*]phosphole[1] (120 mg, 0.22 mmol) in degassed dichloromethane (20 ml) was stirred at room temperature in the dark overnight. The solvent was removed under reduced pressure to dryness. Recrystallization from a concentrated dichloromethane solution by layering hexane afforded a white solid.

BzP-AuCl. Yield: 80 mg, 0.10 mmol; 46 %. ^{1}H NMR (400 MHz, $CDCl_3$, 298 K, δ/ppm): Diastereomer A, δ 2.00 (s, 3H, $-CH_3$), 2.14 (s, 3H, $-CH_3$), 7.09 (s, 1H, thienyl), 7.28 (s, 1H, thienyl), 7.32–7.37 (m, 3H, phenyl), 7.40–7.48 (m, 4H, phenyl), 7.51–7.53 (m, 2H, phenyl), 7.59–7.60 (m, 3H, phenyl), 7.63–7.74 (m, 6H, phenyl), 7.94–7.98 (m, 1H, phenyl). Diastereomer B, δ 2.00 (s, 3H, $-CH_3$), 2.32 (s, 3H, $-CH_3$), 7.26 (s, 1H, thienyl), 7.30 (s, 1H, thienyl), 7.32–7.37 (m, 3H, phenyl), 7.40–7.48 (m, 4H, phenyl), 7.51–7.53 (m, 2H, phenyl), 7.59–7.60 (m, 3H, phenyl), 7.63–7.74 (m, 6H, phenyl), 7.94–7.98 (m, 1H, phenyl). ^{31}P{^{1}H} NMR (162 MHz, $CDCl_3$, 298 K, δ/ppm): δ 29.11, 30.81. Diastereomeric ratio (dr) of A/B = 1.53. HRMS (Positive ESI) calcd for $C_{36}H_{28}AuClPS_2$: *m/z* = 787.0719; found: *m/z* = 787.0665 $[M+H]^+$.

The TMS-protected thieno[3,2-*b*]phosphole-containing alkynyl ligand (TMS-ThP) was prepared using a modified literature procedure as described.[19] A mixture of *N*-iodosuccinimide (72 mg, 0.31 mmol) and 2,3-bis(2,5-dimethylthiophen-3-yl)-1-phenylthieno[3,2-*b*]phosphole-1-oxide[2] (130 mg, 0.29 mmol) in chloroform (30 ml) with a catalytic amount of acetic acid was stirred at room temperature in the dark overnight. The solvent was removed under reduced pressure to offer a yellow solid. The yellow solid was added to a mixture of trimethylsilylacetylene (0.2 ml, 1.45 mmol),

bis(triphenylphosphine)palladium(II) dichloride (20 mg, 10 mmol%) and copper(I) iodide (11 mg, 20 mmol%) in diisopropylamine (40 ml) for stirring under reflux condition in the dark overnight. The reaction mixture was cooled to room temperature, followed by dilution using ethyl acetate (50 mL) and filtration to remove the insoluble solid. Deionized water (50 mL) was used to wash the organic layer, which was subsequently dried over anhydrous magnesium sulfate and filtered. The solvent was removed under reduced pressure to dryness. Column chromatography with silica gel (70–230 mesh) was then employed to purify the crude product by using hexane–ethyl acetate mixture as eluent. Recrystallization by layering hexane onto a concentrated dichloromethane solution of the product afforded a yellow solid.

TMS-ThP. Yield: 107 mg, 0.20 mmol; 69 %. ^{1}H NMR (500 MHz, DMSO-d_6, 353 K, δ/ppm): δ 0.24 (s, 9H, –SiMe$_3$), 1.77 (s, 3H, –CH$_3$), 1.94 (s, 3H, –CH$_3$), 2.27 (s, 3H, –CH$_3$), 2.42 (s, 3H, –CH$_3$), 6.53 (s, 1H, thienyl), 6.88 (s, 1H, thienyl), 7.48–7.53 (m, 3H, thienyl and phenyl), 7.57–7.63 (m, 3H, phenyl). ^{31}P{^{1}H} NMR (203 MHz, DMSO-d_6, 353 K, δ/ppm): δ 26.6. HRMS (Positive ESI) calcd for $C_{29}H_{30}OPS_3Si$: *m*/*z* = 549.0960; found: *m*/*z* = 549.0956 $[M+H]^+$.

The benzo[*b*]phosphole thieno[3,2-*b*]phosphole-containing alkynylgold(I) complex **1** was prepared using a modified literature procedure[19] for the synthesis of related benzo[*b*]phosphole alkynylgold(I) complexes. The benzo[*b*]phosphole chlorogold(I) precursor (BzP-AuCl) (142 mg, 0.18 mmol), TMS-protected thieno[3,2-*b*]phosphole-containing alkyne (TMS-ThP) (100 mg, 0.18 mmol), tetra-*n*-butylammonium fluoride (TBAF) (0.50 ml, 1 M in THF) and sodium hydroxide (20 mg, 0.50 mmol) in dichloromethane solution (50 ml) were stirred at room temperature in the dark overnight. Deionized water (50 mL) was employed to wash the organic layer, which was subsequently dried and filtered. The solvent was removed under reduced pressure to dryness. Column chromatography with silica gel (70–230 mesh) was then employed to purify the crude product by using hexane–ethyl acetate mixture as eluent. Complex **1** was recrystallized by the layering of hexane onto a concentrated dichloromethane solution of the complex, followed by filtration and washing with hexane and methanol. The complex was then dried in air.

Complex **1**. Yield: 75 mg, 0.12 mmol; 67 %. ^{1}H NMR (500 MHz, DMSO-d_6, 343 K, δ/ppm): Diastereomer A, δ 1.76 (s, 3H, –CH$_3$ of thieno[3,2-*b*]phosphole), 1.94 (s, 3H, –CH$_3$ of thieno[3,2-*b*]phosphole), 1.95 (s, 3H, –CH$_3$ of benzo[*b*]phosphole), 2.27 (s, 3H, –CH$_3$ of thieno[3,2-*b*]phosphole and benzo[*b*]phosphole), 2.42 (s, 3H, –CH$_3$ of thieno[3,2-*b*]phosphole), 6.52 (s, 1H, thienyl of thieno[3,2-*b*]phosphole), 6.86 (s, 1H, thienyl of thieno[3,2-*b*]phosphole), 7.05 (s, 1H, thienyl of benzo[*b*]phosphole), 7.23–7.28 (m, 2H, thienyl and phenyl of benzo[*b*]phosphole), 7.31–7.38 (m, 4H, phenyl of benzo[*b*]phophole), 7.40–7.43 (m, 2H, thienyl of thieno[3,2-*b*]phosphole and phenyl of benzo[*b*]phosphole), 7.45–7.50 (m, 5H, phenyl of benzo[*b*]phosphole), 7.50–7.54 (s, 10H, phenyl of thieno[3,2-*b*]phosphole and benzo[*b*]phosphole),

7.68–7.71 (m, 2H, phenyl of benzo[*b*]phosphole), 7.95–7.97 (m, 1H, phenyl of benzo[*b*]phosphole); Diastereomer B, δ 1.76 (s, 3H, $-CH_3$ of thieno[3,2-*b*]phosphole), 1.93 (s, 3H, $-CH_3$ of benzo[*b*]phosphole), 1.94 (s, 3H, $-CH_3$ of thieno[3,2-*b*]phosphole), 2.27 (s, 6H, $-CH_3$ of thieno[3,2-*b*]phosphole and benzo[*b*]phosphole), 2.42 (s, 3H, $-CH_3$ of thieno[3,2-*b*]phosphole), 6.52 (s, 1H, thienyl of thieno[3,2-*b*]phosphole), 6.86 (s, 1H, thienyl of thieno[3,2-*b*]phosphole), 7.23–7.28 (m, 2H, thienyl and phenyl of benzo[*b*]phosphole), 7.31–7.38 (m, 5H, thienyl and phenyl of benzo[*b*]phophole), 7.40–7.43 (m, 2H, thienyl of thieno[3,2-*b*]phosphole and phenyl of benzo[*b*]phosphole), 7.45–7.50 (m, 5H, phenyl of benzo[*b*]phosphole), 7.50–7.54 (s, 10H, phenyl of thieno[3,2-*b*]phosphole and benzo[*b*]phosphole), 7.68–7.71 (m, 2H, phenyl of benzo[*b*]phosphole), 7.95–7.97 (m, 1H, phenyl of benzo[*b*]phosphole). $^{31}P\{^{1}H\}$ NMR (203 MHz, DMSO-d_6, 343 K, δ/ppm): δ 29.41 (thieno[3,2-*b*]phosphole), 46.94 (benzo[*b*]phosphole), 47.08 (benzo[*b*]phosphole). Diastereomeric ratio (dr) of A/B = 1.25. HRMS (Positive ESI) calcd for $C_{63}H_{48}AuOP_2S_5$ *m*/*z* = 1227.1444; found 1227.1350 $[M+H]^+$. Elemental analyses calcd (%) for $C_{63}H_{47}AuOP_2S_5 \cdot H_2O$: C 59.80, H 3.97; found (%): C 59.52, H 3.73. The water molecules are likely to be introduced from traces of water in methanol as well as during the filtration and washing steps due to water condensation from the moist air to give the hydrate.

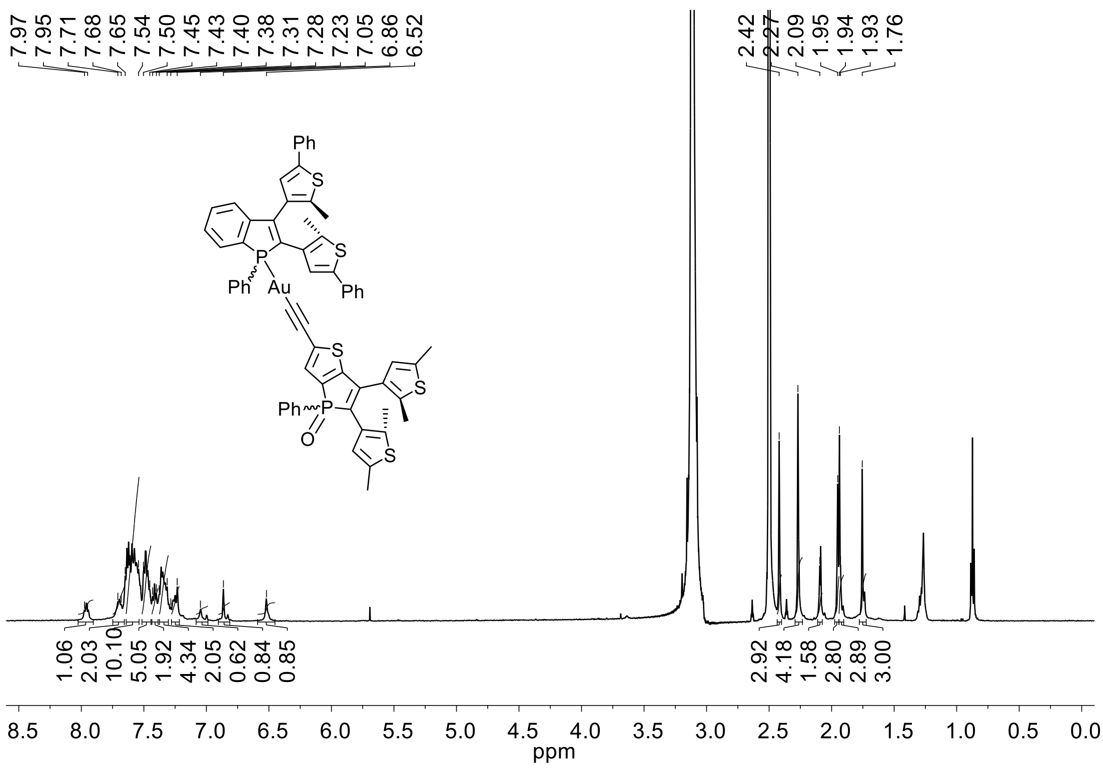

Supplementary Figure 1. ^{1}H NMR spectrum of complex **1** in DMSO-d_6 at 343 K.

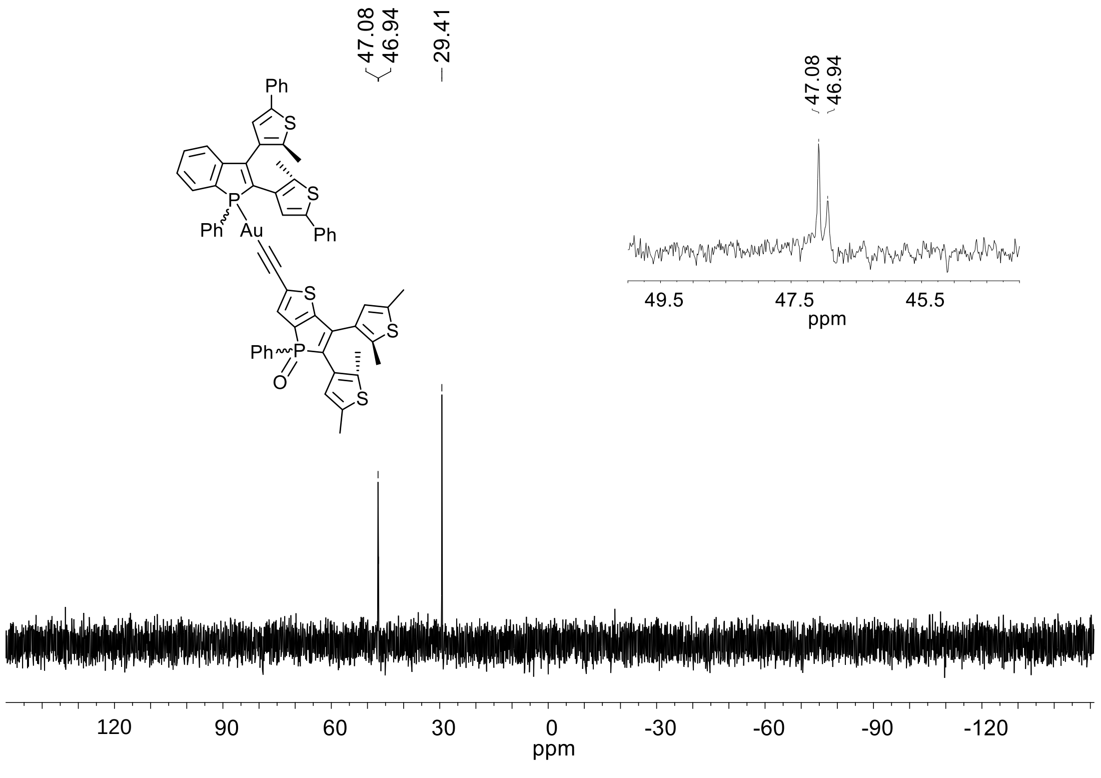

Supplementary Figure 2. ^{31}P{^{1}H} NMR spectrum of complex **1** in DMSO-d_6 at 343 K.

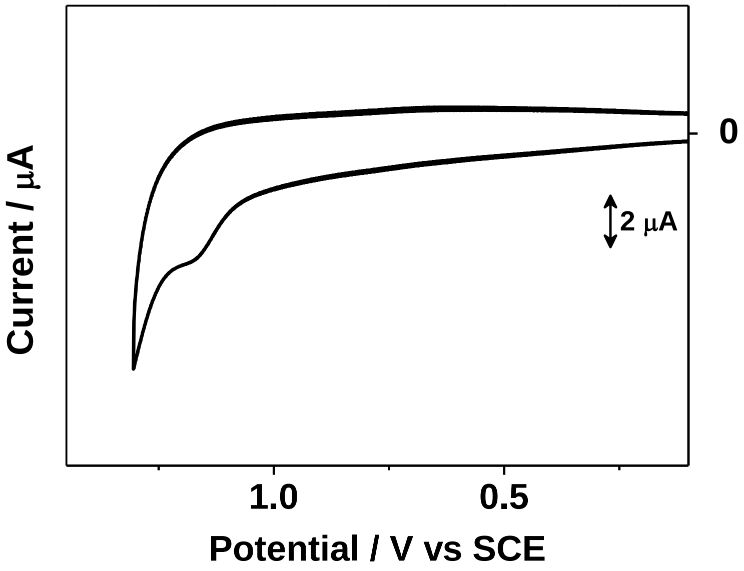

Supplementary Figure 3. Cyclic voltammogram of complex **1** showing the oxidative scan.

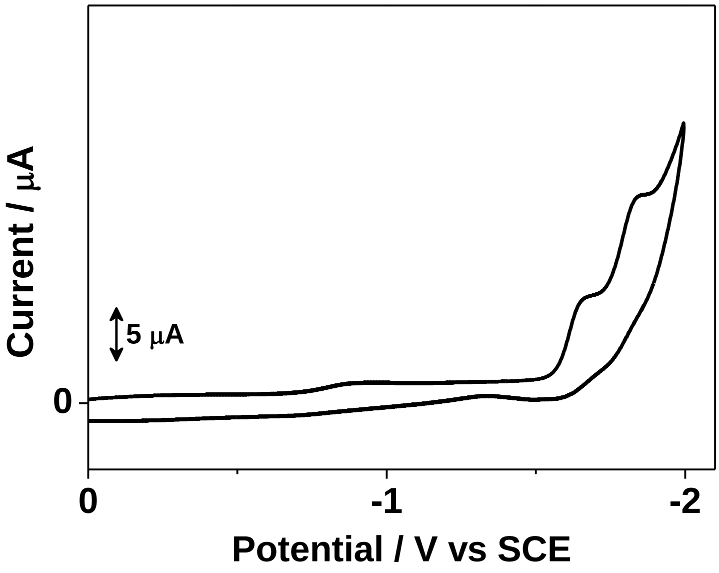

Supplementary Figure 4. Cyclic voltammogram of complex **1** showing the reductive scan.

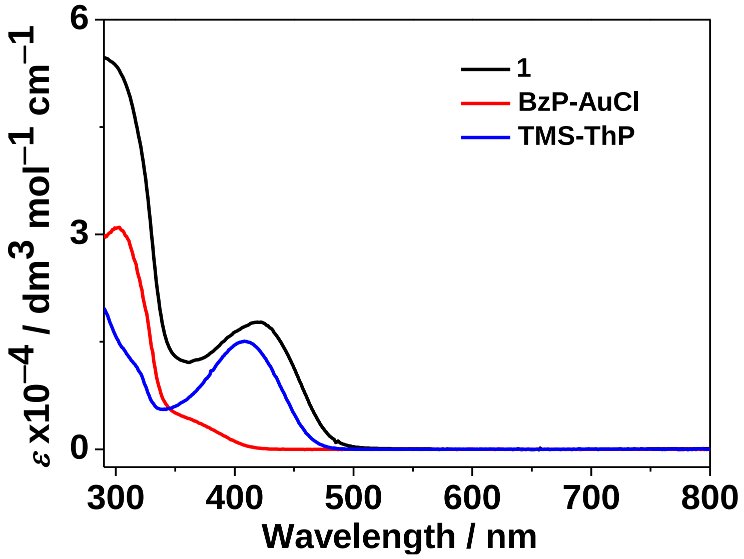

Supplementary Figure 5. Electronic absorption spectra of complex **1** and its benzo[*b*]phosphole chlorogold(I) precursor (BzP-AuCl) and TMS-protected thieno[3,2-*b*]phosphole-containing alkyne (TMS-ThP).

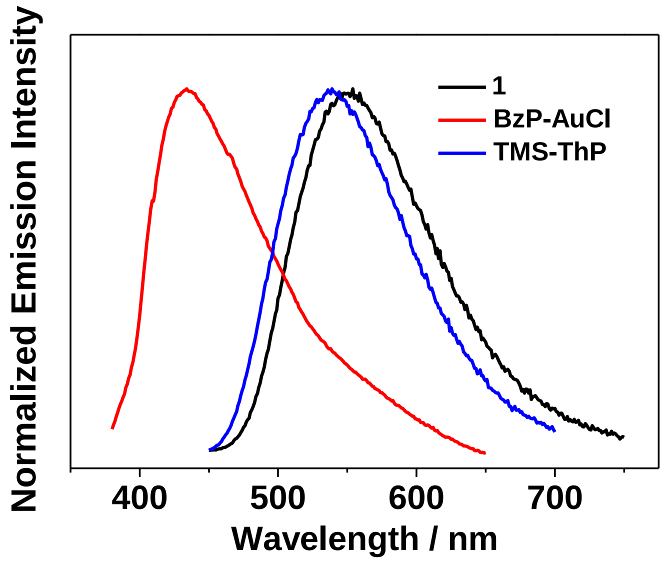

Supplementary Figure 6. Emission spectra of complex **1** and its benzo[*b*]phosphole chlorogold(I) precursor (BzP-AuCl) and TMS-protected thieno[3,2-*b*]phosphole-containing alkyne (TMS-ThP).

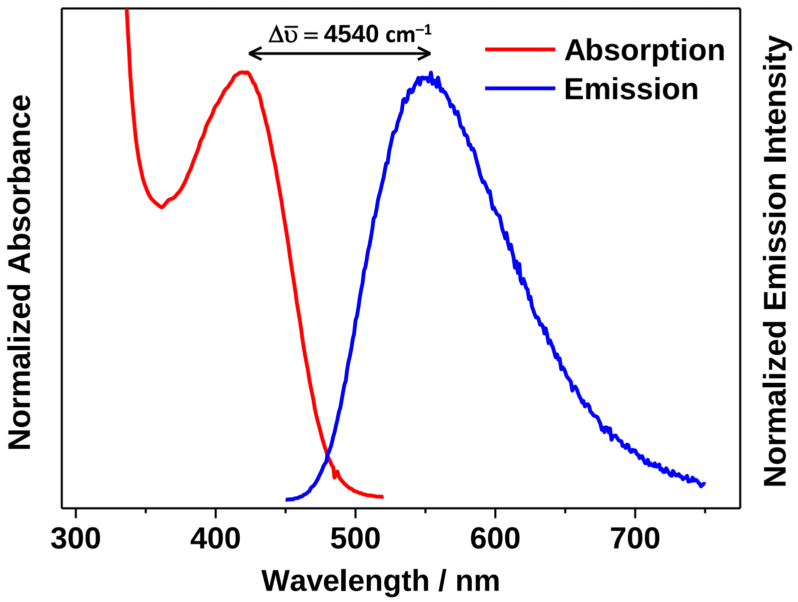

Supplementary Figure 7. Electronic absorption and emission spectra of complex **1** with Stokes shift.

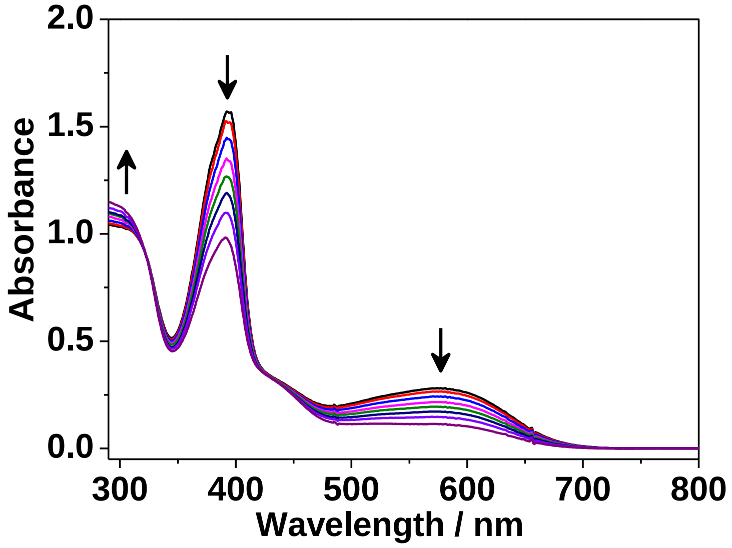

Supplementary Figure 8. Photocycloreversion reaction of **1-cc** to form **1-oc** upon excitation of visible light at *ca*. 580 nm.

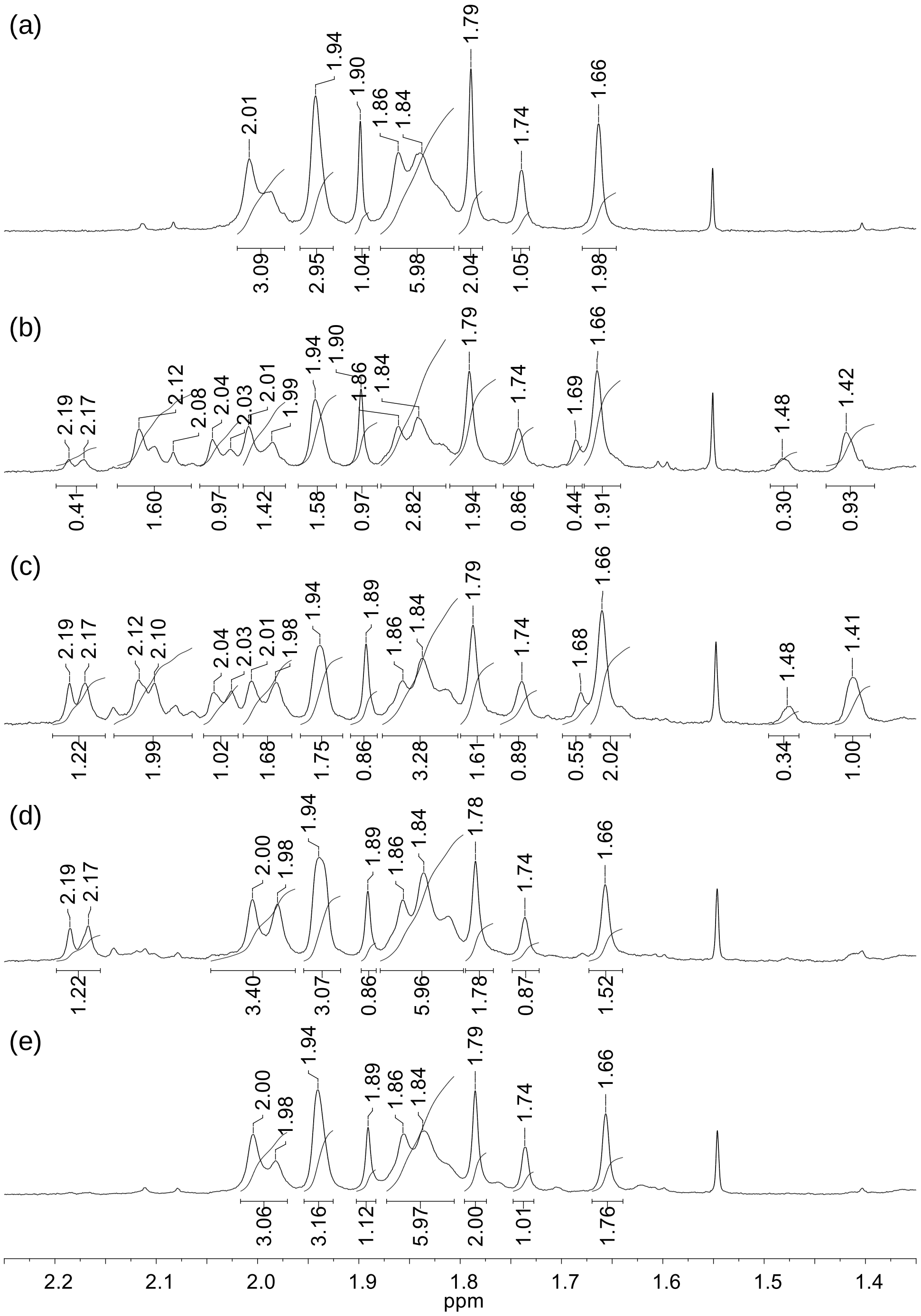

Supplementary Figure 9. ^{1}H NMR spectra of complex **1** (8×10^{-4} M, 500 MHz, 298 K) (a) before photoirradiation, (b) upon photoexcitation at *ca*. 440 nm for photocyclization of the thieno[3,2-*b*]phosphole moiety, (c) upon subsequent UV excitation at *ca*. 300 nm for photocyclization of the benzo[*b*]phosphole moiety, (d) upon subsequent photoexcitation at *ca*. 500 nm for photocycloreversion of the thieno[3,2-*b*]phosphole moiety, and (e) eventually subjected to photoirradiation at 580 nm for the photocycloreversion of the benzo[*b*]phosphole moiety in non-degassed benzene-d_6.

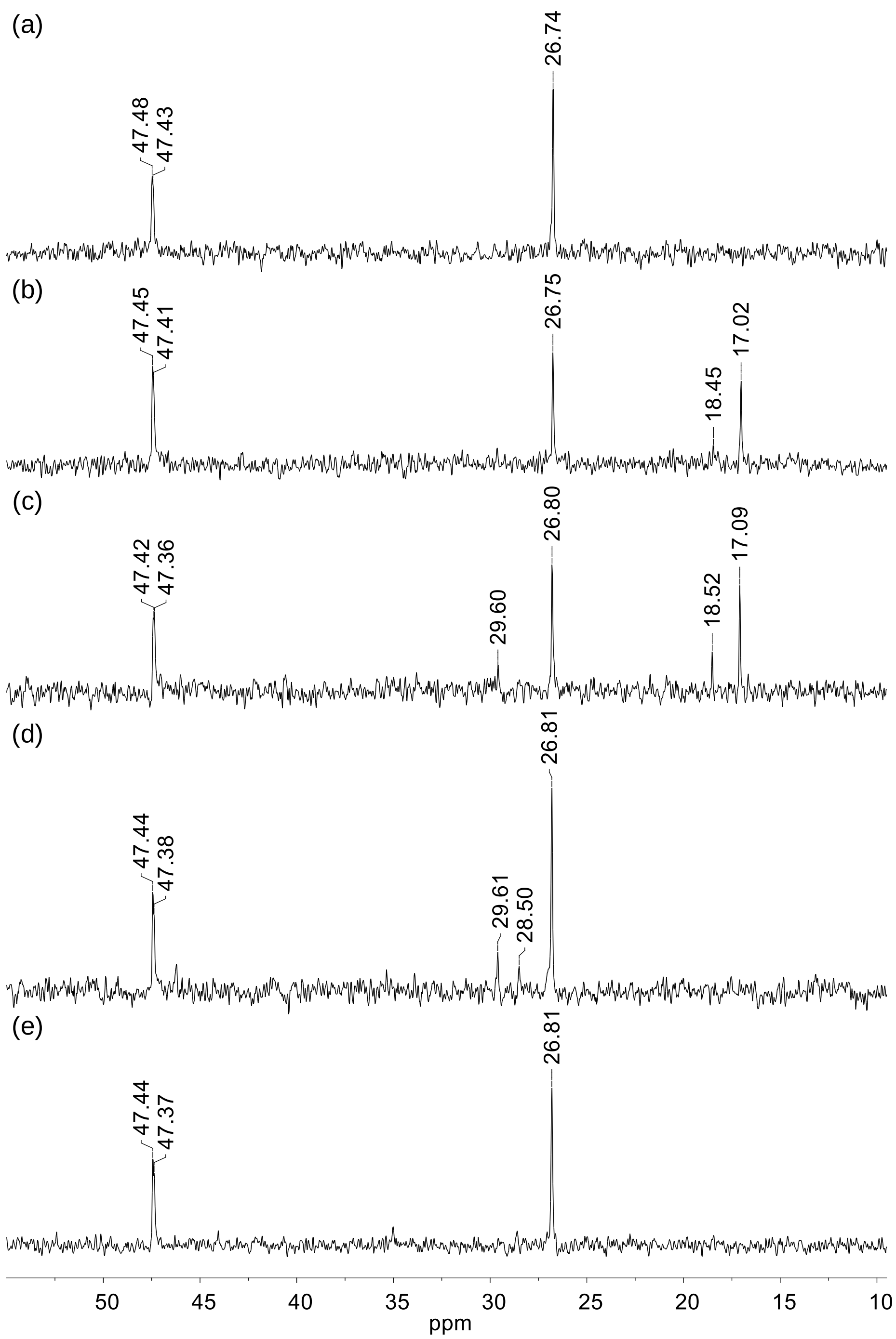

Supplementary Figure 10. $^{31}P\{^{1}H\}$ NMR spectra of complex **1** (8×10^{-4} M, 203 MHz, 298 K) (a) before photoirradiation, (b) upon photoexcitation at *ca*. 440 nm for photocyclization of the thieno[3,2-*b*]-phosphole moiety, (c) upon subsequent UV excitation at *ca*. 300 nm for photocyclization of the benzo[*b*]phosphole moiety, (d) upon subsequent photoexcitation at *ca*. 500 nm for photocycloreversion of the thieno[3,2-*b*]phosphole moiety, and (e) eventually subjected to photoirradiation at 580 nm for the photocycloreversion of the benzo[*b*]phosphole moiety in non-degassed benzene-d_6.

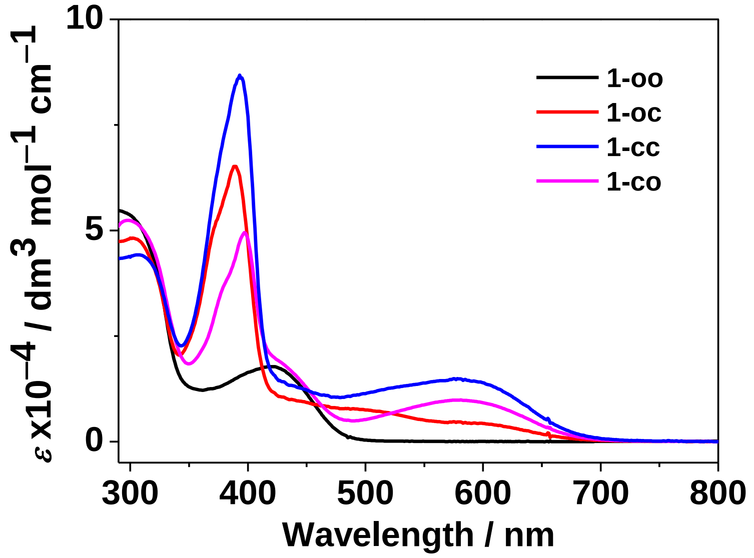

Supplementary Figure 11. Electronic absorption spectra of the open form and the photogenerated closed forms of complex **1** including the open-open (**1-oo**), open-closed (**1-oc**), closed-closed (**1-cc**) and closed-open (**1-co**) forms.

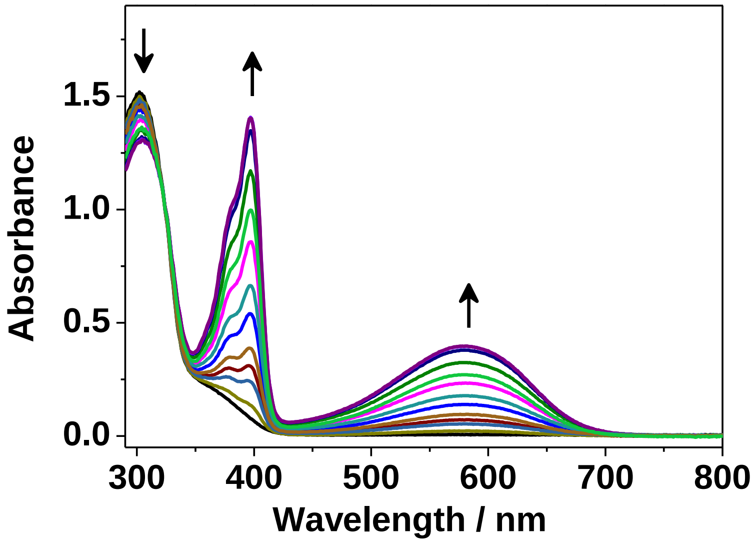

Supplementary Figure 12. UV−Vis absorption spectral changes of benzo[*b*]phosphole chlorogold(I) precursor (BzP-AuCl) in degassed benzene solution upon UV excitation at *ca*. 300 nm.

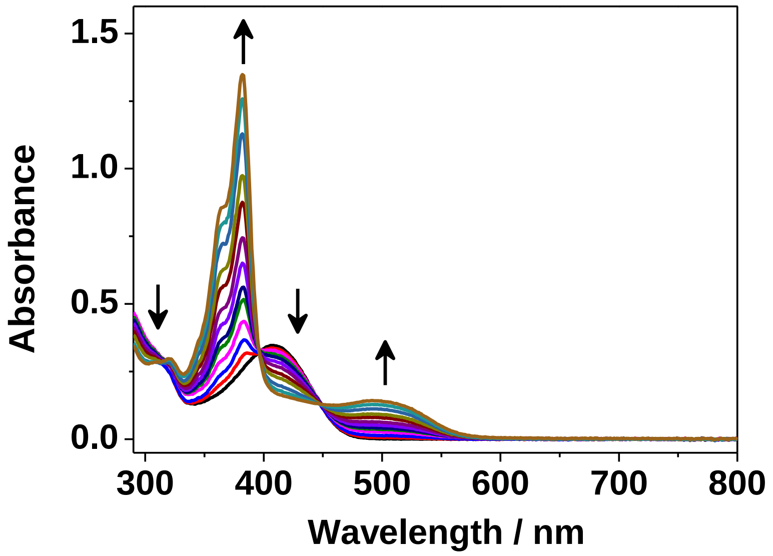

Supplementary Figure 13. UV−Vis absorption spectral changes of TMS-protected thieno[3,2-*b*]-phosphole-containing alkyne (TMS-ThP) in degassed benzene solution upon excitation of visible light at *ca.* 440 nm.

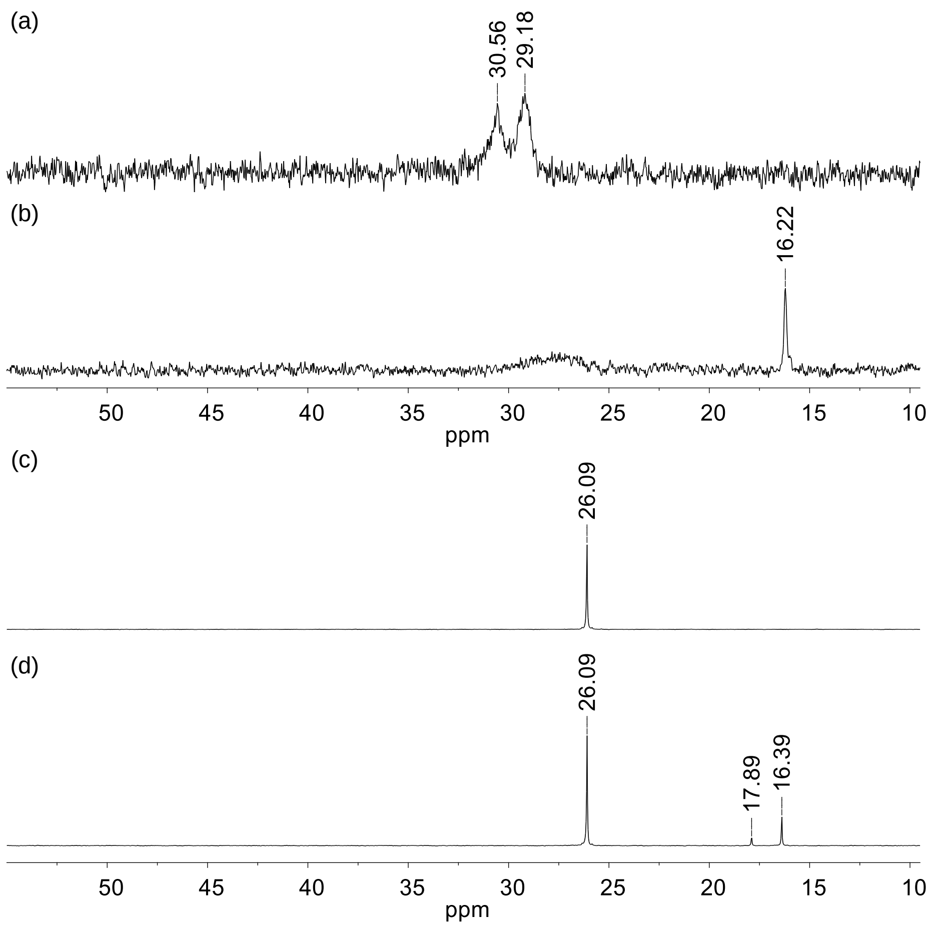

Supplementary Figure 14. $^{31}P\{^{1}H\}$ NMR spectra of BzP-AuCl (1×10^{-2} M, 203 MHz, 298 K) (a) before photoirradiation, and (b) upon UV excitation at *ca*. 300 nm for photocyclization of the benzo[*b*]phosphole moiety in benzene-d_6. $^{31}P\{^{1}H\}$ NMR spectra of TMS-ThP (2×10^{-2} M, 203 MHz, 298 K) (c) before photoirradiation, and (d) upon photoexcitation with visible light at *ca*. 440 nm for photocyclization of the thieno[3,2-*b*]phosphole moiety in benzene-d_6.

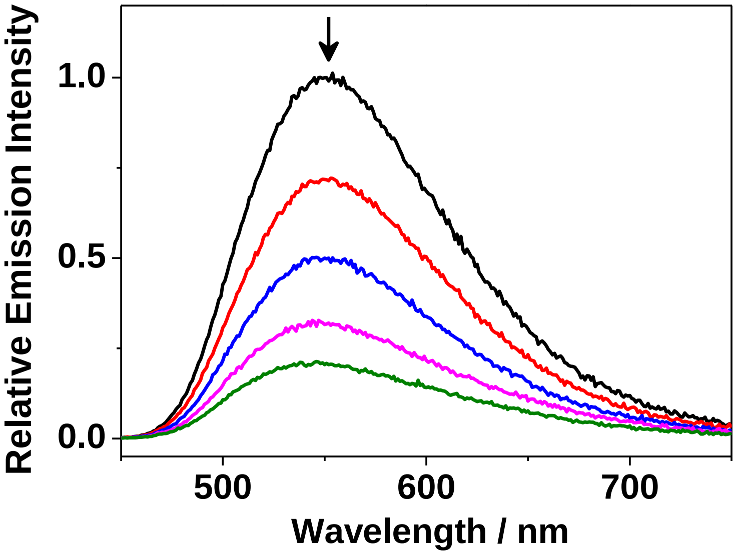

Supplementary Figure 15. Emission spectral changes of complex **1** in degassed benzene solution upon excitation of visible light at *ca*. 411 nm.

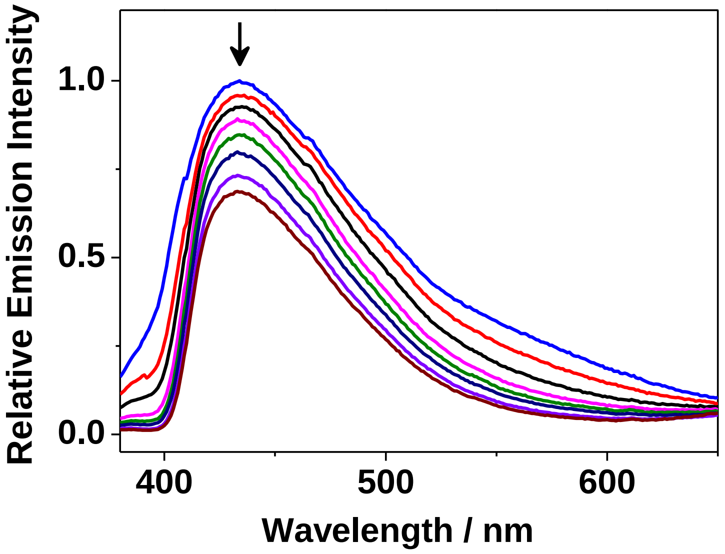

Supplementary Figure 16. Emission spectral changes of benzo[*b*]phosphole chlorogold(I) precursor (BzP-AuCl) in degassed benzene solution upon UV excitation at *ca*. 300 nm.

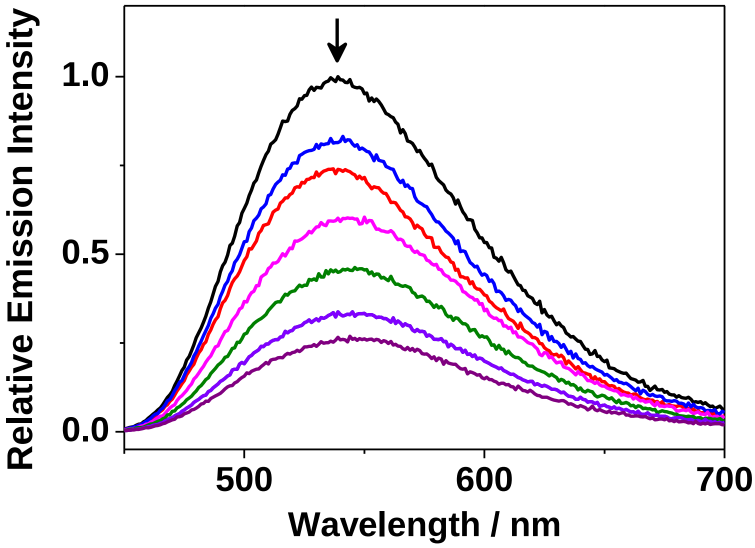

Supplementary Figure 17. Emission spectral changes of TMS-protected thieno[3,2-*b*]phosphole-containing alkyne (TMS-ThP) in degassed benzene solution upon excitation of visible light at *ca.* 397 nm.

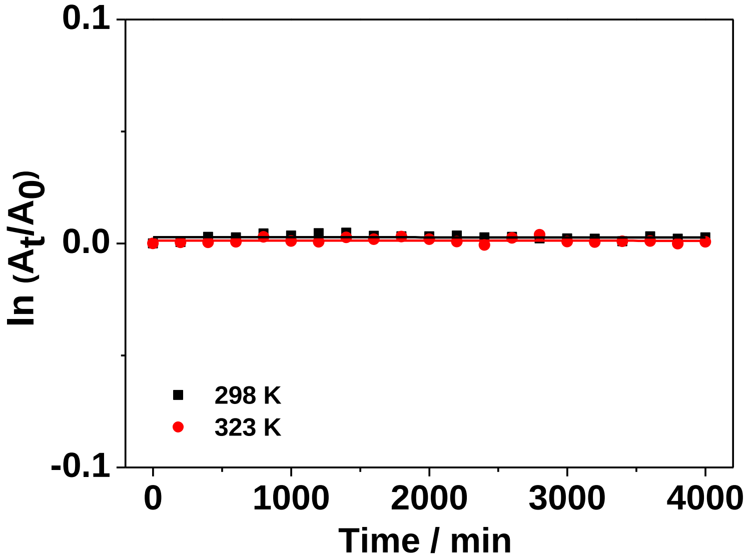

Supplementary Figure 18. A plot of ln(A_t/A_0) versus time for the absorbance decay at 580 nm of the closed form of the benzo[*b*]phosphole moiety of complex **1** at 25 and 50 °C in nitrogen-flushed toluene solution. A_0 and A_t denote initial absorbance and absorbance at time t, respectively; solid lines represent theoretical linear fits.

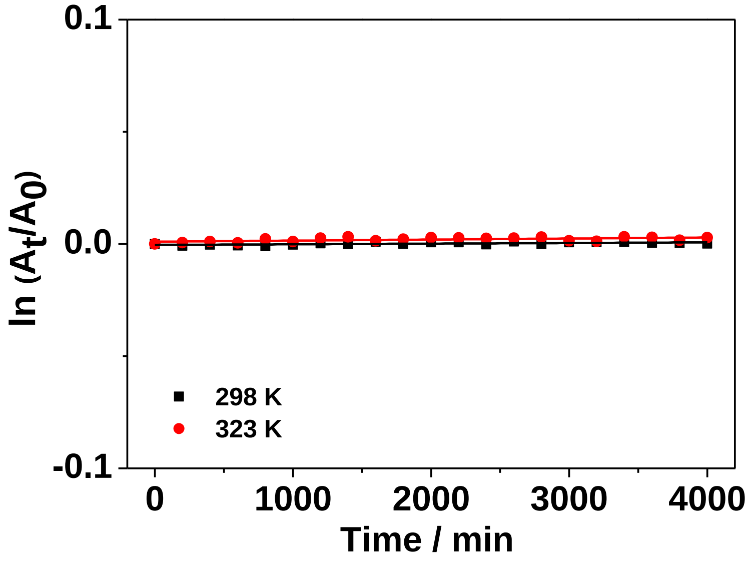

Supplementary Figure 19. A plot of ln(A_t/A_0) versus time for the absorbance decay at 500 nm of the closed form of the thieno[3,2-*b*]phosphole moiety of complex **1** at 25 and 50 °C in nitrogen-flushed toluene solution. A_0 and A_t denote initial absorbance and absorbance at time t, respectively; solid lines represent theoretical linear fits.

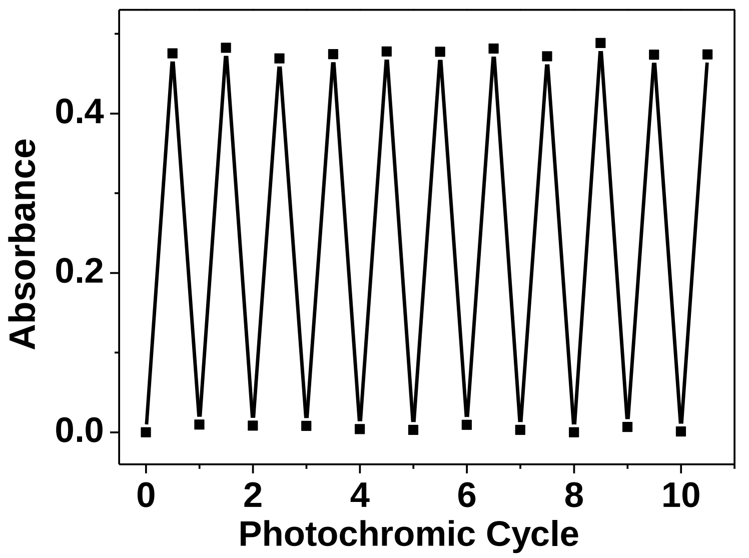

Supplementary Figure 20. Fatigue resistance studies of the benzo[*b*]phosphole moiety of complex **1** in non-degassed benzene solutions under ambient conditions over ten photochromic cycles; the UV–vis absorption changes are monitored at 580 nm upon alternate photoirradiation at 300 nm and 550 nm.

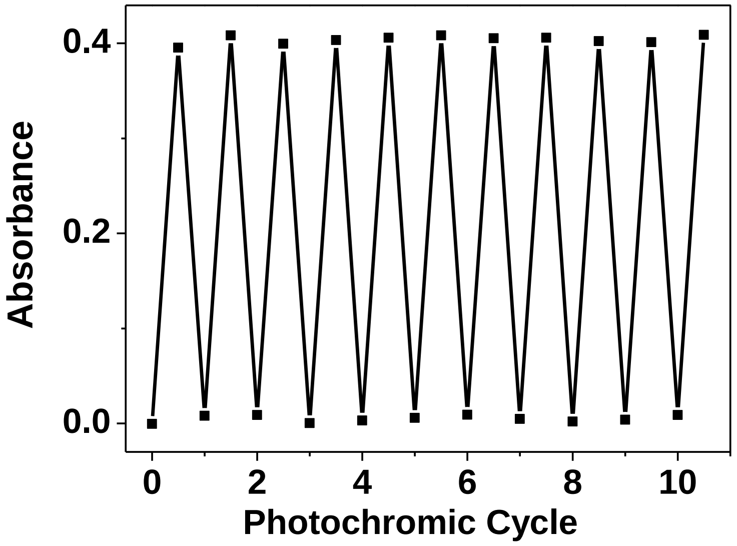

Supplementary Figure 21. Fatigue resistance studies of the thieno[3,2-*b*]phosphole moiety of complex **1** in non-degassed benzene solutions under ambient conditions over ten photochromic cycles; the UV–vis absorption changes are monitored at 500 nm upon alternate photoirradiation at 440 nm and 550 nm.

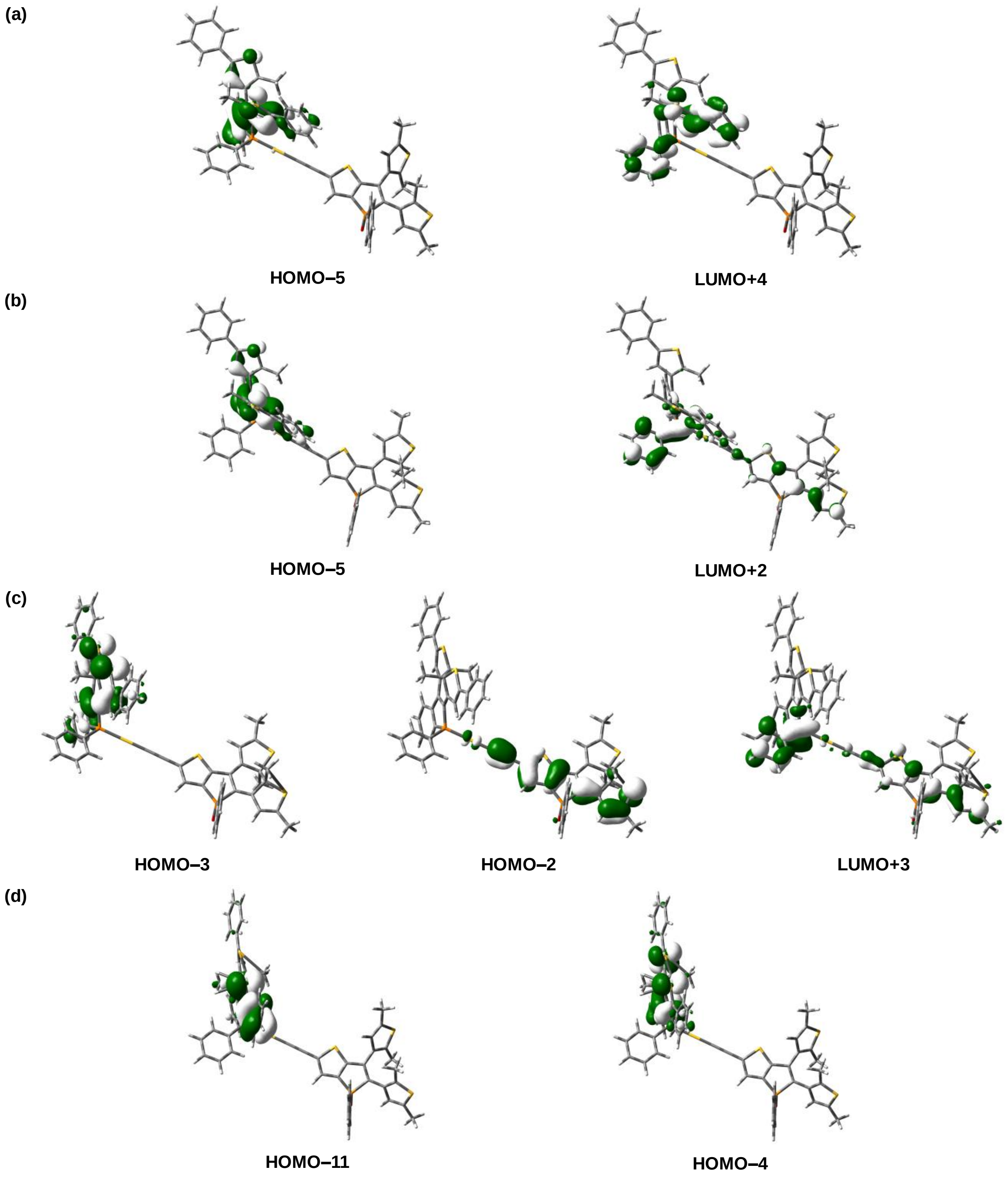

Supplementary Figure 22. Spatial plots (isovalue = 0.03) of selected molecular orbitals of (a) **1-oo**, (b) **1-oc**, (c) **1-cc**, and (d) **1-co** at the optimized ground-state geometry.

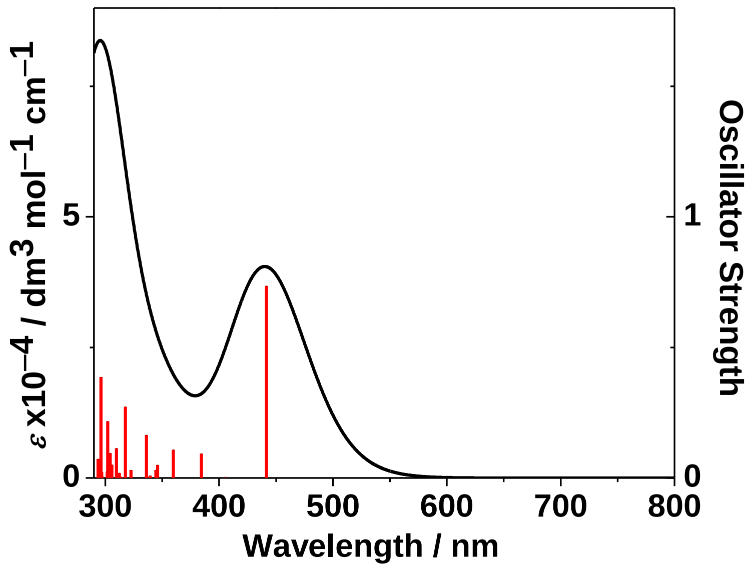

Supplementary Figure 23. Simulated UV–vis spectrum of **1-oo** computed by TDDFT/CPCM using benzene as the solvent. The peaks of the vertical transitions were broadened with Gaussian function.

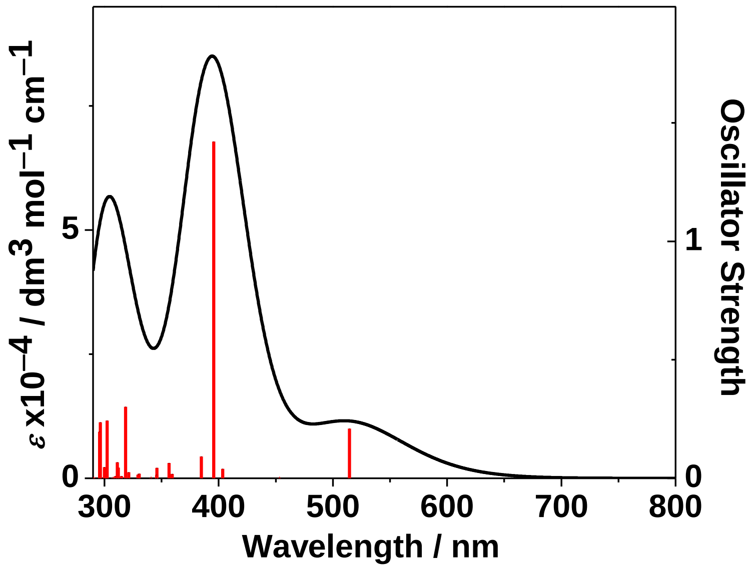

Supplementary Figure 24. Simulated UV–vis spectrum of **1-oc** computed by TDDFT/CPCM using benzene as the solvent. The peaks of the vertical transitions were broadened with Gaussian function.

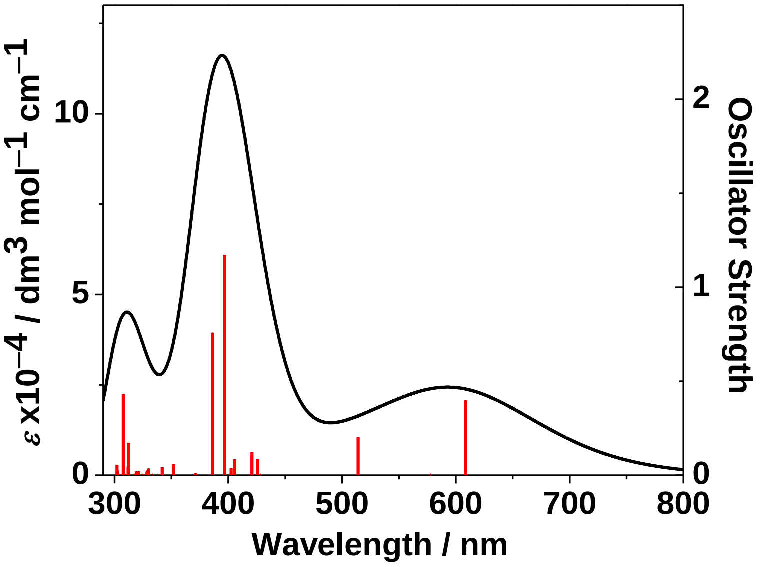

Supplementary Figure 25. Simulated UV–vis spectrum of **1-cc** computed by TDDFT/CPCM using benzene as the solvent. The peaks of the vertical transitions were broadened with Gaussian function.

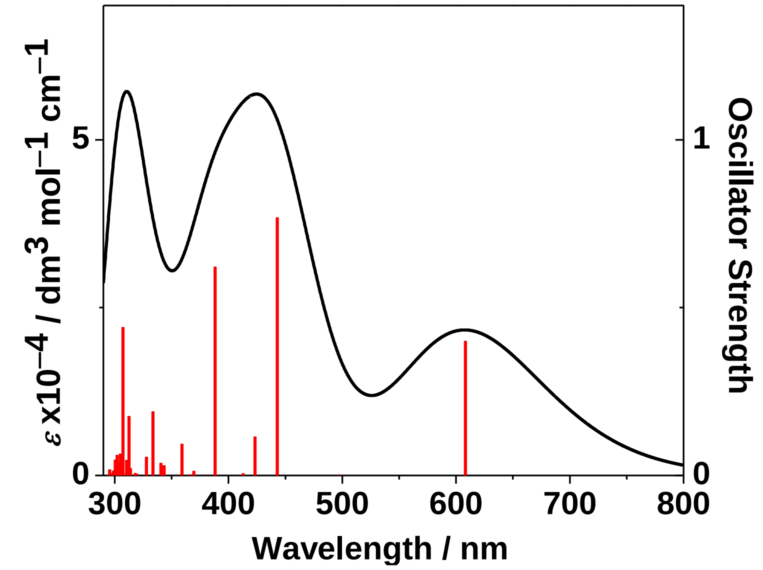

Supplementary Figure 26. Simulated UV–vis spectrum of **1-co** computed by TDDFT/CPCM using benzene as the solvent. The peaks of the vertical transitions were broadened with Gaussian function.

Supplementary Table 1. Electrochemical data of complex **1**[a].

Complex	Oxidation [$E_{pa}{}^{b}$/ V *vs.* SCE]	Reduction [$E_{pc}{}^{c}$/ V *vs.* SCE],
**1**	[+1.17]	[−1.66], [−1.85]

[a]In 0.1 M $^{n}Bu_4NPF_6$ dichloromethane solutions; working electrode, glassy carbon; scan rate, 100 mVs^{-1}. [b]E_{pa} is reported for irreversible oxidation wave. [c]E_{pc} is reported for irreversible reduction wave.

Supplementary Table 2. Photophysical data of complex **1**.

Configuration	Absorption λ_{max} / nm (ε / dm^3 mol^{-1} cm^{-1})	Emission λ_{em} / nm^b (τ_o/μs)	$\phi_{lum}{}^{c}$
**1-oo**	291sh (55000), 419 (17800)	550 (< 0.1)	0.033
**1-oc**	302 (48200), 388 (65200), 501 (5460)		
**1-co**	298 (52400), 397 (49500), 428 (18800), 585 (9780)		
**1-cc**	306 (44200), 393 (86800), 500sh (11300), 580 (14900)		

[a]Data obtained in degassed benzene solution at 298 K. [b]Emission maxima are corrected values. [c]Quinine sulfate in 0.5 M H_2SO_4 is used as the standard for the determination of relative luminescence quantum yield.

Supplementary Table 3. Photochromic data of complex **1**[a].

Photoswitch	Photochromic Quantum Yield / ϕ^{b}		Conversion at Photostationary State (PSS) (%)[c]
	Photocyclization	Photocycloreversion	
Benzo[*b*]phosphole	0.29[c]	0.011[d]	90
Thieno[3,2-*b*]phosphole	0.64[e]	0.082[f]	87

[a]Data obtained in degassed benzene solution at 298 K with an uncertainty of ±10 %. [b]Photochromic quantum yields are measured using ferrioxalate as the chemical actinometer. [c]302 nm was used as the excitation wavelength. [d]550 nm was used as the excitation wavelength. [e]436 nm was used as the excitation wavelength. [f]500 nm was used as the excitation wavelength.

Supplementary Table 4. The first twenty singlet excited states (S_n) of **1-oo** computed by TDDFT/CPCM using benzene as the solvent.

Complex	S_n	Excitation[a] (Coefficient)[b]	Vertical excitation wavelength / nm	f^c
**1-oo**	S_1	H→L+1 (0.70)	441	0.736
	S_2	H→L (0.69)	405	0.000
	S_3	H–1→L (0.70)	384	0.094
	S_4	H–2→L+1 (0.69)	359	0.108
	S_5	H–3→L (0.68)	346	0.050
	S_6	H–4→L+1 (0.68)	344	0.031
	S_7	H–1→L+1 (0.68)	339	0.009
	S_8	H→L+2 (0.65)	336	0.165
	S_9	H–2→L (0.62)	322	0.031
	S_{10}	H–5→L (0.64)	317	0.273
	S_{11}	H→L+3 (0.52)	314	0.003
		H→L+4 (–0.37)		
	S_{12}	H→L+4 (0.53)	312	0.020
		H→L+3 (0.39)		
	S_{13}	H–1→L+2 (0.67)	310	0.114
	S_{14}	H–6→L+1 (0.55)	305	0.051
	S_{15}	H–3→L+1 (0.62)	304	0.024
	S_{16}	H→L+5 (0.42)	304	0.096
	S_{17}	H–1→L+3 (0.67)	302	0.217
	S_{18}	H–4→L (0.49)	302	0.022
		H→L+7 (0.31)		
	S_{19}	H–4→L (0.49)	302	0.024
		H→L+7 (–0.32)		
	S_{20}	H–7→L+1 (0.40)	297	0.006
		H→L+7 (0.35)		

[a]Orbitals involved in the major excitation (H = HOMO and L = LUMO).
[b]The coefficients in the configuration interaction (CI) expansion that are less than 0.3 are not listed.
[c]Oscillator strengths.

Supplementary Table 5. The first twenty singlet excited states (S_n) of **1-oc** computed by TDDFT/CPCM using benzene as the solvent.

Complex	S_n	Excitation[a] (Coefficient)[b]	Vertical excitation wavelength / nm	f[c]
**1-oc**	S_1	H→L (0.69)	514	0.209
	S_2	H→L+1 (0.69)	453	0.001
	S_3	H→L+2 (0.42)	403	0.040
		H→L+4 (–0.41)		
		H–1→L (–0.35)		
	S_4	H–1→L (0.58)	395	1.421
		H→L+2 (0.32)		
	S_5	H–2→L+1 (0.68)	385	0.092
	S_6	H–1→L+1 (0.58)	359	0.018
	S_7	H→L+4 (0.45)	356	0.065
		H→L+2 (0.40)		
		H–1→L+1 (–0.33)		
	S_8	H–3→L+1 (0.65)	346	0.044
	S_9	H–2→L (0.66)	345	0.015
	S_{10}	H→L+3 (0.68)	345	0.003
	S_{11}	H→L+5 (0.69)	340	0.001
	S_{12}	H→L+8 (0.52)	330	0.020
	S_{13}	H–4→L (0.59)	329	0.016
	S_{14}	H→L+6 (0.62)	321	0.025
	S_{15}	H–5→L+1 (0.65)	318	0.301
	S_{16}	H→L+7 (0.46)	315	0.010
		H→L+14 (0.34)		
	S_{17}	H–1→L+2 (0.53)	312	0.045
		H–1→L+4 (–0.34)		
	S_{18}	H–2→L+2 (0.56)	311	0.067
	S_{19}	H–3→L (0.62)	310	0.010
	S_{20}	H→L+14 (0.53)	307	0.002
		H→L+8 (0.30)		

[a]Orbitals involved in the major excitation (H = HOMO and L = LUMO).

[b]The coefficients in the configuration interaction (CI) expansion that are less than 0.3 are not listed.

[c]Oscillator strengths.

Supplementary Table 6. The first twenty singlet excited states (S_n) of **1-cc** computed by TDDFT/CPCM using benzene as the solvent.

Complex	S_n	Excitation[a] (Coefficient)[b]	Vertical excitation wavelength / nm	f^c
**1-cc**	S_1	H–1→L (0.70)	608	0.399
	S_2	H→L (0.70)	577	0.001
	S_3	H→L+1 (0.70)	514	0.203
	S_4	H–2→L (0.69)	428	0.003
	S_5	H–1→L+1 (0.53)	426	0.085
		H–1→L+2 (–0.42)		
	S_6	H–1→L+2 (0.49)	420	0.122
		H–1→L+1 (0.45)		
	S_7	H→L+2 (0.57)	405	0.084
	S_8	H–2→L+1 (0.45)	402	0.038
		H→L+2 (–0.36)		
	S_9	H–2→L+1 (0.44)	397	1.172
		H→L+3 (0.35)		
		H–3→L (–0.31)		
	S_{10}	H–3→L (0.55)	386	0.758
	S_{11}	H–1→L+3 (0.60)	371	0.010
		H–1→L+4 (0.32)		
	S_{12}	H→L+4 (0.55)	351	0.059
		H→L+3 (0.39)		
	S_{13}	H–1→L+5 (0.57)	342	0.042
		H–1→L+4 (0.36)		
	S_{14}	H–4→L (0.69)	337	0.000
	S_{15}	H→L+5 (0.47)	333	0.004
		H→L+7 (–0.37)		
	S_{16}	H–4→L+1 (0.57)	330	0.036
	S_{17}	H–1→L+6 (0.51)	329	0.028
	S_{18}	H→L+7 (0.48)	328	0.020
		H–2→L+2 (0.32)		
	S_{19}	H–2→L+2 (0.53)	325	0.007
		H→L+5 (–0.39)		
	S_{20}	H–1→L+4 (0.40)	321	0.021

[a]Orbitals involved in the major excitation (H = HOMO and L = LUMO).

[b]The coefficients in the configuration interaction (CI) expansion that are less than 0.3 are not listed.

[c]Oscillator strengths.

Supplementary Table 7. The first twenty singlet excited states (S_n) of **1-co** computed by TDDFT/CPCM using benzene as the solvent.

Complex	S_n	Excitation[a] (Coefficient)[b]	Vertical excitation wavelength / nm	f^c
**1-co**	S_1	H→L (0.71)	608	0.401
	S_2	H–1→L (0.70)	497	0.000
	S_3	H–1→L+1 (0.69)	443	0.769
	S_4	H→L+2 (0.59)	423	0.116
	S_5	H→L+1 (0.64)	413	0.006
	S_6	H–4→L (0.63)	388	0.622
	S_7	H–2→L (0.70)	379	0.000
	S_8	H→L+3 (0.68)	319	0.014
	S_9	H–1→L+2 (0.69)	365	0.003
	S_{10}	H–2→L+1 (0.69)	359	0.094
	S_{11}	H–3→L (0.70)	354	0.000
	S_{12}	H–3→L+1 (0.69)	343	0.030
	S_{13}	H→L+4 (0.68)	340	0.038
	S_{14}	H–1→L+3 (0.64)	333	0.191
	S_{15}	H→L+5 (0.64)	328	0.056
	S_{16}	H–5→L (0.52)	320	0.004
		H–6→L (0.44)		
	S_{17}	H→L+9 (0.40)	318	0.008
		H→L+7 (0.36)		
	S_{18}	H–6→L (0.50)	314	0.022
		H–5→L (–0.45)		
	S_{19}	H–9→L (0.61)	312	0.177
	S_{20}	H–1→L+4 (0.55)	310	0.046

[a]Orbitals involved in the major excitation (H = HOMO and L = LUMO).

[b]The coefficients in the configuration interaction (CI) expansion that are less than 0.3 are not listed.

[c]Oscillator strengths.

Supplementary Table 8. Relative Gibbs free energies, distances between the reactive carbon atoms, and the transitions responsible for the photocyclization reaction of **1-oo**, **1-oc**, **1-cc** and **1-co** optimized at the PBE0 level.

Isomer	ΔG (kcal mol^{-1})	d_1 (Å)	d_2 (Å)	Transitions responsible for photocyclization
**1-oo**	0.00	3.618	3.562	H→L+1
**1-oc**	0.67	3.615	1.536	H–5→L+1
**1-cc**	11.90	1.534	1.536	–
**1-co**	10.71	1.534	3.563	H–1→L+1

Supplementary Table 9. Cartesian coordinates of the optimized ground-state geometry of **1-oo**.

1	C	-5.498734	0.756523	-2.278665	54	H	-4.711533	1.634780	2.519871
2	C	-4.561268	-0.102676	-2.883342	55	H	-4.061006	2.095700	0.946724
3	C	-4.290141	-0.039173	-4.243613	56	H	-4.687996	3.340257	2.041941
4	C	-4.979576	0.889130	-5.024250	57	C	-10.055729	2.725914	0.473982
5	C	-5.898537	1.755571	-4.433381	58	C	-10.515984	3.939717	1.004923
6	C	-6.156678	1.703820	-3.064721	59	C	-10.993253	1.843778	-0.085032
7	C	-5.637202	0.565490	-0.819253	60	C	-11.868325	4.261208	0.975950
8	C	-4.825058	-0.400499	-0.301671	61	H	-9.806118	4.645844	1.427987
9	H	-3.552360	-0.697414	-4.693457	62	C	-12.342693	2.173474	-0.123666
10	H	-4.788632	0.944917	-6.091493	63	H	-10.661552	0.885234	-0.473231
11	H	-6.415787	2.488478	-5.045561	64	C	-12.787857	3.382400	0.408123
12	H	-6.858876	2.397524	-2.613023	65	H	-12.202980	5.207366	1.391299
13	P	-3.776695	-1.121308	-1.612087	66	H	-13.052229	1.476226	-0.559978
14	C	-4.354482	-2.835366	-1.829654	67	H	-13.843541	3.635568	0.383101
15	C	-3.453583	-3.892701	-1.678349	68	C	0.465514	-0.649155	-1.192453
16	C	-5.696616	-3.098815	-2.129380	69	C	1.682256	-0.516774	-1.083120
17	C	-3.893664	-5.206962	-1.823392	70	C	3.072520	-0.349461	-0.957817
18	H	-2.411226	-3.685775	-1.449386	71	C	5.360067	-0.727511	-0.965042
19	C	-6.129055	-4.411232	-2.275266	72	C	5.340721	0.573763	-0.506684
20	H	-6.398098	-2.277720	-2.253530	73	C	7.687569	0.414963	-0.540980
21	C	-5.227983	-5.465541	-2.121394	74	S	3.734619	1.173394	-0.392516
22	H	-3.190558	-6.025926	-1.705466	75	C	4.081677	-1.258320	-1.225753
23	H	-7.169751	-4.613778	-2.510271	76	H	3.879984	-2.255887	-1.596414
24	H	-5.569381	-6.490107	-2.236541	77	P	7.085866	-1.221559	-1.123243
25	Au	-1.490214	-0.873985	-1.380817	78	C	6.630521	1.242546	-0.281582
26	C	-4.743841	-0.909893	1.068209	79	C	6.671947	2.656533	0.111148
27	C	-3.503979	-1.051359	1.769402	80	C	7.417694	3.622249	-0.528625
28	C	-5.821851	-1.353009	1.810210	81	C	5.880715	3.191448	1.183480
29	C	-3.630391	-1.570358	3.030660	82	S	7.150306	5.168238	0.204538
30	H	-2.553843	-0.729732	1.354831	83	C	6.034264	4.533072	1.371767
31	S	-5.302417	-1.916558	3.358097	84	H	5.238682	2.583377	1.812866
32	C	-2.581051	-1.833398	4.016995	85	C	9.119033	0.636122	-0.371903
33	C	-2.860506	-1.872683	5.390670	86	C	9.725626	1.215632	0.724572
34	C	-1.259234	-2.050336	3.597000	87	C	10.071973	0.187494	-1.351579
35	C	-1.851476	-2.119903	6.314799	88	S	11.445351	1.234397	0.520725
36	H	-3.873810	-1.690369	5.739583	89	C	11.368642	0.448083	-1.026848
37	C	-0.251262	-2.284977	4.524231	90	H	9.773987	-0.309847	-2.269054
38	H	-1.025069	-2.051689	2.536236	91	C	8.331365	3.491933	-1.699911
39	C	-0.541653	-2.323339	5.887024	92	H	9.370562	3.345447	-1.383261
40	H	-2.089503	-2.144629	7.374330	93	H	8.050160	2.622170	-2.299117
41	H	0.764920	-2.450529	4.178089	94	H	8.292127	4.379345	-2.337822
42	H	0.246724	-2.513084	6.609368	95	C	5.391602	5.408586	2.395360
43	C	-7.262689	-1.423110	1.432950	96	H	6.130869	5.879118	3.052392
44	H	-7.808032	-0.525581	1.746385	97	H	4.804195	6.209674	1.934232
45	H	-7.359935	-1.502592	0.346892	98	H	4.718417	4.812895	3.016906
46	H	-7.751618	-2.290639	1.884974	99	C	9.113208	1.740289	1.978718
47	C	-6.561331	1.403740	-0.037602	100	H	8.895675	2.812622	1.910261
48	C	-7.950116	1.539076	-0.352041	101	H	8.165997	1.229254	2.172204
49	C	-6.188024	2.165476	1.050051	102	H	9.770015	1.582538	2.839025
50	C	-8.634049	2.375475	0.491016	103	C	12.603237	0.146369	-1.809203
51	H	-8.420450	1.027698	-1.184932	104	H	13.290071	-0.501289	-1.253714
52	S	-7.548217	3.021168	1.685109	105	H	12.331983	-0.365618	-2.735800
53	C	-4.843208	2.321613	1.676067	106	H	13.152011	1.056571	-2.074428

107	C	7.447947	-2.391702	0.216833
108	C	8.184115	-3.534619	-0.100404
109	C	7.024919	-2.174421	1.532432
110	C	8.495794	-4.458458	0.895026
111	H	8.502533	-3.687838	-1.127590
112	C	7.339125	-3.098190	2.522649
113	H	6.447432	-1.287012	1.780223
114	C	8.074596	-4.240320	2.203869
115	H	9.067427	-5.348081	0.647020
116	H	7.009643	-2.930128	3.543863
117	H	8.317859	-4.960895	2.979570
118	O	7.583029	-1.707780	-2.455763

Supplementary Table 10. Cartesian coordinates of the optimized ground-state geometry of **1-oc**.

1	C	-5.618360	0.496674	-2.312985	54	H	-4.716488	2.006531	2.303313
2	C	-4.682844	-0.421818	-2.826705	55	H	-4.116553	2.271834	0.666302
3	C	-4.449275	-0.534065	-4.190742	56	H	-4.737056	3.636940	1.611163
4	C	-5.174817	0.274446	-5.066175	57	C	-10.131448	2.752613	0.286147
5	C	-6.092501	1.198400	-4.567722	58	C	-10.597022	4.020020	0.665557
6	C	-6.313173	1.323448	-3.197221	59	C	-11.069039	1.792795	-0.125292
7	C	-5.714046	0.497567	-0.837635	60	C	-11.954632	4.318028	0.632689
8	C	-4.872302	-0.381611	-0.221820	61	H	-9.887650	4.784479	0.971973
9	H	-3.712806	-1.236032	-4.571253	62	C	-12.424152	2.097686	-0.168859
10	H	-5.013260	0.192231	-6.136554	63	H	-10.732375	0.795598	-0.393219
11	H	-6.638409	1.837425	-5.255548	64	C	-12.874535	3.360574	0.211572
12	H	-7.014925	2.060979	-2.820475	65	H	-12.293411	5.306489	0.929182
13	P	-3.849788	-1.256711	-1.456355	66	H	-13.133639	1.340194	-0.489483
14	C	-4.410307	-2.990197	-1.432686	67	H	-13.934441	3.595057	0.183191
15	C	-3.490019	-4.009892	-1.176317	68	C	0.398129	-0.724548	-1.196606
16	C	-5.757044	-3.303903	-1.652721	69	C	1.617522	-0.591514	-1.122008
17	C	-3.915309	-5.336391	-1.137360	70	C	3.010306	-0.426037	-1.034496
18	H	-2.444262	-3.764065	-1.009690	71	C	5.299961	-0.821103	-1.036612
19	C	-6.174777	-4.628591	-1.615057	72	C	5.299290	0.529156	-0.715520
20	H	-6.473808	-2.512982	-1.858542	73	C	7.651881	0.161476	-0.642915
21	C	-5.254280	-5.644956	-1.356445	74	S	3.692295	1.143548	-0.632439
22	H	-3.197157	-6.126040	-0.938182	75	C	4.008763	-1.361138	-1.220761
23	H	-7.219166	-4.870471	-1.788404	76	H	3.795359	-2.390825	-1.480718
24	H	-5.584232	-6.679307	-1.328117	77	P	6.987075	-1.438068	-1.189318
25	Au	-1.560705	-0.969040	-1.315705	78	C	6.586465	1.167639	-0.535551
26	C	-4.742038	-0.702023	1.200545	79	C	6.898341	2.478977	-0.326111
27	C	-3.479142	-0.732265	1.873674	80	C	8.373114	2.884772	-0.386906
28	C	-5.789425	-1.053502	2.030239	81	C	6.046888	3.608317	-0.103148
29	C	-3.558234	-1.076688	3.196975	82	S	8.434143	4.550125	0.421755
30	H	-2.547456	-0.459115	1.388403	83	C	6.690156	4.736329	0.288202
31	S	-5.213580	-1.395736	3.622414	84	H	4.966536	3.576588	-0.199822
32	C	-2.474678	-1.189331	4.174910	85	C	8.931504	0.460244	-0.309889
33	C	-2.711499	-1.048041	5.549875	86	C	10.117207	-0.338064	-0.416787
34	C	-1.162132	-1.439984	3.744662	87	S	11.042521	2.047657	0.160157
35	C	-1.670218	-1.153255	6.465269	88	C	11.270400	0.343484	-0.212271
36	H	-3.717129	-0.836063	5.904217	89	H	10.101837	-1.386432	-0.696544
37	C	-0.122089	-1.532325	4.661464	90	C	8.755918	3.071654	-1.864925
38	H	-0.959958	-1.579682	2.686509	91	H	8.670748	2.121222	-2.399767
39	C	-0.370207	-1.391902	6.025921	92	H	8.072514	3.790698	-2.322707
40	H	-1.875472	-1.039030	7.525787	93	H	9.777976	3.442698	-1.965066
41	H	0.886256	-1.726566	4.307441	94	C	6.078359	6.054289	0.610893
42	H	0.443385	-1.470365	6.740923	95	H	6.331429	6.367863	1.629988
43	C	-7.239968	-1.190351	1.713250	96	H	6.445256	6.834038	-0.066038
44	H	-7.789112	-0.264634	1.919957	97	H	4.990698	6.002125	0.524491
45	H	-7.369611	-1.417668	0.651744	98	C	8.900566	1.696535	1.833253
46	H	-7.701339	-1.993771	2.294293	99	H	7.834836	1.496601	1.978237
47	C	-6.630340	1.419610	-0.146240	100	H	9.473043	0.866349	2.253948
48	C	-8.029068	1.494261	-0.436018	101	H	9.161786	2.612860	2.365969
49	C	-6.241088	2.322754	0.820899	102	C	12.655956	-0.195110	-0.289107
50	C	-8.704276	2.425497	0.308940	103	H	13.169843	-0.090597	0.672882
51	H	-8.512625	0.871462	-1.180810	104	H	12.640540	-1.251840	-0.564397
52	S	-7.598059	3.236854	1.376520	105	H	13.248358	0.349874	-1.032303
53	C	-4.883172	2.577645	1.382917	106	C	7.272279	-2.631911	0.150285

107	C	7.823593	-3.871722	-0.180797
108	C	6.954801	-2.343611	1.481938
109	C	8.059257	-4.817500	0.814859
110	H	8.060874	-4.078841	-1.220540
111	C	7.190846	-3.289960	2.473119
112	H	6.522484	-1.380558	1.742447
113	C	7.743540	-4.526766	2.139532
114	H	8.488169	-5.781153	0.555417
115	H	6.943755	-3.064951	3.506610
116	H	7.926754	-5.264466	2.915730
117	O	7.432202	-1.992737	-2.513135
118	C	9.214343	1.819171	0.332230

Supplementary Table 11. Cartesian coordinates of the optimized ground-state geometry of **1-cc**.

1	C	-5.652784	0.273466	-2.533743	54	H	-4.000732	1.600494	1.021666
2	C	-4.663265	-0.625459	-2.989725	55	H	-4.983201	3.077109	1.086521
3	C	-4.420354	-0.825323	-4.344457	56	H	-4.670444	2.153700	2.572110
4	C	-5.190836	-0.142806	-5.281169	57	C	-8.847272	4.073712	0.336992
5	C	-6.197175	0.721544	-4.849150	58	C	-9.124116	4.855279	1.469349
6	C	-6.431924	0.931491	-3.494511	59	C	-9.494485	4.391027	-0.869444
7	C	-5.728202	0.375191	-1.068744	60	C	-10.005773	5.927454	1.393986
8	C	-4.889553	-0.631368	-0.417946	61	H	-8.627564	4.631431	2.409314
9	H	-3.642557	-1.511741	-4.667341	62	C	-10.372134	5.463904	-0.941396
10	H	-5.015343	-0.290509	-6.342162	63	H	-9.325690	3.777494	-1.749050
11	H	-6.812921	1.238095	-5.579646	64	C	-10.631364	6.238249	0.189370
12	H	-7.243524	1.584247	-3.198865	65	H	-10.201592	6.523798	2.280181
13	P	-3.810282	-1.450518	-1.616319	66	H	-10.865404	5.691238	-1.881875
14	C	-4.297982	-3.208410	-1.673753	67	H	-11.321882	7.074360	0.131669
15	C	-3.311674	-4.198224	-1.697040	68	C	0.412410	-0.796138	-1.240215
16	C	-5.650013	-3.571853	-1.697951	69	C	1.625263	-0.623329	-1.141615
17	C	-3.674651	-5.542921	-1.744607	70	C	3.009318	-0.410842	-1.024572
18	H	-2.262139	-3.913692	-1.675963	71	C	5.313502	-0.712018	-1.032506
19	C	-6.006873	-4.913980	-1.744619	72	C	5.255024	0.607358	-0.605622
20	H	-6.420037	-2.804938	-1.678584	73	C	7.620215	0.331209	-0.538089
21	C	-5.019478	-5.899846	-1.767967	74	S	3.623640	1.147889	-0.491816
22	H	-2.904839	-6.308649	-1.762209	75	C	4.046866	-1.287152	-1.273019
23	H	-7.056144	-5.193466	-1.762675	76	H	3.877696	-2.300589	-1.616009
24	H	-5.302159	-6.947951	-1.803758	77	P	7.025546	-1.246022	-1.215206
25	Au	-1.535192	-1.100721	-1.413233	78	C	6.513285	1.281243	-0.361945
26	C	-4.955389	-0.878551	0.918597	79	C	6.769002	2.583166	-0.045478
27	C	-4.150967	-1.731142	1.727973	80	C	8.225955	3.052818	-0.059115
28	C	-6.060849	-0.191433	1.710688	81	C	5.869555	3.655105	0.258377
29	C	-4.341036	-1.583139	3.070986	82	S	8.211885	4.649031	0.880688
30	H	-3.392523	-2.385744	1.312614	83	C	6.462371	4.773573	0.744935
31	S	-5.554397	-0.370902	3.480013	84	H	4.792098	3.586648	0.149074
32	C	-3.640467	-2.294998	4.137815	85	C	8.884016	0.654543	-0.169720
33	C	-3.618612	-1.792687	5.447789	86	C	10.102504	-0.083782	-0.327785
34	C	-2.970419	-3.500629	3.870016	87	S	10.924432	2.284305	0.444946
35	C	-2.938334	-2.466315	6.455103	88	C	11.225147	0.625681	-0.058800
36	H	-4.123017	-0.857578	5.676426	89	H	10.132208	-1.106430	-0.689524
37	C	-2.290584	-4.169868	4.878221	90	C	8.610343	3.373336	-1.513500
38	H	-3.000667	-3.925514	2.871511	91	H	8.568662	2.466106	-2.123286
39	C	-2.270420	-3.655706	6.174454	92	H	7.900262	4.097886	-1.919023
40	H	-2.927889	-2.058634	7.461446	93	H	9.616541	3.793192	-1.573584
41	H	-1.780754	-5.101991	4.653733	94	C	5.793902	6.035090	1.165886
42	H	-1.740049	-4.182780	6.961915	95	H	6.026842	6.276040	2.209266
43	C	-7.361935	-0.989133	1.517061	96	H	6.132190	6.881179	0.557144
44	H	-7.668284	-0.960333	0.467447	97	H	4.709956	5.945204	1.064823
45	H	-7.190547	-2.029132	1.804723	98	C	8.788651	1.713750	2.064434
46	H	-8.170089	-0.582954	2.128167	99	H	7.731075	1.460483	2.183287
47	C	-6.395349	1.283246	-0.287334	100	H	9.391853	0.876139	2.422879
48	C	-7.248813	2.374679	-0.637699	101	H	9.009422	2.594350	2.670632
49	C	-6.142412	1.261608	1.225343	102	C	12.632232	0.151657	-0.164602
50	C	-7.913994	2.950229	0.404927	103	H	13.135307	0.200250	0.807496
51	H	-7.344387	2.758696	-1.644460	104	H	12.662071	-0.879666	-0.522670
52	S	-7.563433	2.190218	1.955365	105	H	13.206359	0.777450	-0.856748
53	C	-4.863250	2.073340	1.500618	106	C	7.349996	-2.528975	0.029907

107	C	7.979260	-3.704338	-0.386335
108	C	6.989160	-2.369347	1.372093
109	C	8.249995	-4.713562	0.535366
110	H	8.248100	-3.812167	-1.433425
111	C	7.260053	-3.378918	2.289251
112	H	6.495290	-1.457303	1.698435
113	C	7.891141	-4.550727	1.870920
114	H	8.739589	-5.626912	0.209753
115	H	6.978733	-3.253934	3.330890
116	H	8.101516	-5.337991	2.589410
117	O	7.505035	-1.676144	-2.572965
118	C	9.106159	1.968402	0.580924

Supplementary Table 12. Cartesian coordinates of the optimized ground-state geometry of **1-co**.

1	C	-5.563072	0.180695	-2.595528	54	H	-3.986723	1.572868	0.968703
2	C	-4.562094	-0.722861	-3.015970	55	H	-4.973973	3.047544	0.988845
3	C	-4.291263	-0.945080	-4.361892	56	H	-4.688634	2.150354	2.495893
4	C	-5.044527	-0.281106	-5.325534	57	C	-8.826063	4.018800	0.144961
5	C	-6.061671	0.587480	-4.928787	58	C	-9.125062	4.821201	1.256912
6	C	-6.324284	0.819908	-3.582970	59	C	-9.452571	4.310698	-1.078672
7	C	-5.668342	0.307260	-1.134307	60	C	-10.008074	5.889073	1.145113
8	C	-4.840206	-0.685457	-0.449578	61	H	-8.644623	4.617039	2.209646
9	H	-3.505071	-1.634334	-4.657245	62	C	-10.331666	5.379336	-1.186982
10	H	-4.847208	-0.446337	-6.380088	63	H	-9.266517	3.680650	-1.942993
11	H	-6.663831	1.089689	-5.680303	64	C	-10.612953	6.174632	-0.076220
12	H	-7.143204	1.475456	-3.315027	65	H	-10.221189	6.501915	2.015927
13	P	-3.734092	-1.520960	-1.611690	66	H	-10.808770	5.586934	-2.140277
14	C	-4.214230	-3.281422	-1.648151	67	H	-11.304524	7.007421	-0.162347
15	C	-3.224022	-4.267508	-1.635738	68	C	0.477157	-0.834218	-1.170691
16	C	-5.564222	-3.650583	-1.691594	69	C	1.686560	-0.646709	-1.058737
17	C	-3.581108	-5.614263	-1.666938	70	C	3.067477	-0.418329	-0.928206
18	H	-2.176153	-3.978337	-1.599865	71	C	5.370496	-0.687789	-0.948812
19	C	-5.915220	-4.994725	-1.721850	72	C	5.291042	0.594168	-0.444781
20	H	-6.337221	-2.886456	-1.699856	73	C	7.642941	0.545172	-0.480880
21	C	-4.923942	-5.976885	-1.709462	74	S	3.658594	1.113182	-0.309778
22	H	-2.808329	-6.377136	-1.656824	75	C	4.117990	-1.268646	-1.227941
23	H	-6.962927	-5.278678	-1.754867	76	H	3.962692	-2.261070	-1.633084
24	H	-5.202044	-7.026574	-1.732461	77	P	7.117003	-1.095456	-1.121456
25	Au	-1.465070	-1.155933	-1.371191	78	C	6.548690	1.313408	-0.194503
26	C	-4.932708	-0.910346	0.889286	79	C	6.524587	2.713088	0.247994
27	C	-4.143027	-1.747194	1.729168	80	C	7.224601	3.734585	-0.355733
28	C	-6.055630	-0.212975	1.647118	81	C	5.708876	3.172211	1.336977
29	C	-4.360767	-1.576983	3.065293	82	S	6.884966	5.239589	0.431128
30	H	-3.375061	-2.407134	1.340529	83	C	5.799379	4.511991	1.572859
31	S	-5.584515	-0.360762	3.429098	84	H	5.095519	2.512910	1.942906
32	C	-3.681012	-2.269422	4.158079	85	C	9.062871	0.825444	-0.301862
33	C	-3.683573	-1.743145	5.458778	86	C	9.642419	1.393671	0.814913
34	C	-3.007241	-3.480180	3.925180	87	C	10.035397	0.454324	-1.294654
35	C	-3.023189	-2.398611	6.491011	88	S	11.359663	1.497221	0.614891
36	H	-4.191382	-0.803698	5.660508	89	C	11.318897	0.761817	-0.958863
37	C	-2.347335	-4.131268	4.958236	90	H	9.760358	-0.024309	-2.229110
38	H	-3.019338	-3.923333	2.934255	91	C	8.143705	3.688750	-1.529076
39	C	-2.351265	-3.593403	6.244960	92	H	9.188934	3.584509	-1.215626
40	H	-3.031525	-1.972606	7.489761	93	H	7.906551	2.826058	-2.156830
41	H	-1.834441	-5.067796	4.760655	94	H	8.059948	4.593452	-2.137792
42	H	-1.836586	-4.106375	7.051910	95	C	5.115407	5.319764	2.624954
43	C	-7.350559	-1.017574	1.441192	96	H	5.831139	5.802491	3.298993
44	H	-7.635834	-1.007588	0.385309	97	H	4.490016	6.106974	2.190639
45	H	-7.182400	-2.052018	1.750021	98	H	4.471736	4.671239	3.224504
46	H	-8.171814	-0.603299	2.028952	99	C	9.006465	1.846568	2.085220
47	C	-6.353781	1.226286	-0.382109	100	H	8.740135	2.909571	2.053161
48	C	-7.203521	2.308547	-0.768179	101	H	8.083483	1.286532	2.259268
49	C	-6.131136	1.231328	1.135465	102	H	9.669598	1.689486	2.940795
50	C	-7.890927	2.899801	0.250963	103	C	12.566019	0.542930	-1.748894
51	H	-7.280471	2.674805	-1.783129	104	H	13.281699	-0.090883	-1.214226
52	S	-7.568859	2.168096	1.821017	105	H	12.318445	0.050214	-2.692426
53	C	-4.859806	2.051299	1.422286	106	H	13.072768	1.485490	-1.982948

```
107  C    7.532210  -2.294717   0.177065
108  C    8.326112  -3.386801  -0.177737
109  C    7.094006  -2.147704   1.497424
110  C    8.680516  -4.329852   0.784833
111  H    8.655238  -3.486047  -1.208165
112  C    7.450925  -3.090377   2.454864
113  H    6.471197  -1.300483   1.774330
114  C    8.244286  -4.181497   2.098516
115  H    9.297108  -5.179927   0.507489
116  H    7.109401  -2.976920   3.479646
117  H    8.520852  -4.917020   2.848622
118  O    7.637307  -1.510895  -2.469113
```

Supplementary Table 13. The SOC constants between the singlet states (S_n) and the energetically close triplet excited states (T_m) in **1-oo**, **1-oc** and **1-co**.

Complex	Singlet excited state S_n (E / eV)	Energetically close triplet excited state T_m (E / eV)	$\langle S_n \vert \hat{H}_{SO} \vert T_m \rangle$ (cm^{-1})
**1-oo**	S_1 (2.76)	T_2 (2.43)	3.73
		T_3 (2.78)	2.24
**1-oc**	S_4 (3.10)	T_8 (2.99)	1.16
		T_9 (3.28)	30.28
**1-co**	S_3 (2.75)	T_4 (2.45)	20.94
		T_5 (2.78)	2.88

Supplementary References

1. Wu, N. M.-W., Wong, H.-L. & Yam, V. W.-W. Photochromic benzo[*b*]phosphole oxide with excellent thermal irreversibility and fatigue resistance in the thin film solid state *via* direct attachment of dithienyl units to the weakly aromatic heterocycle. *Chem. Sci.* **8**, 1309−1315 (2017).
2. Wu, N. M.-W., Ng, M., Lam, W. H., Wong, H.-L. & Yam, V. W.-W. Photochromic heterocycle-fused thieno[*3,2-b*]phosphole oxides as visible light switches without sacrificing photoswitching efficiency. *J. Am. Chem. Soc.* **139**, 15142−15150 (2017).
3. Connelly, N. G. & Geiger, E. W. Chemical redox agents for organometallic chemistry. *Chem. Rev.* **96**, 877−910 (1996).
4. Demas, J. N. & Crosby, G. A. The measurement of photoluminescence quantum yields. *J. Phys. Chem.* **75**, 991−1024(1971).
5. Kuhn, H. J., Braslavsky, S. E. & Schmidt, R. Chemical actinometry (IUPAC technical report). *Pure Appl. Chem.* **76**, 2105−2146 (2004).
6. Frisch, M. J., Trucks, G. W., Schlegel, H. B., Scuseria, G. E., Robb, M. A., Cheeseman, J. R., Scalmani, G. Barone, V. Mennucci, B. Petersson, G. A., Nakatsuji, H., Caricato, M., Li, X., Hratchian, H. P., Izmaylov, A. F., Bloino, J., Zheng, G., Sonnenberg, J. L., Hada, M., Ehara, M., Toyota, K., Fukuda, R., Hasegawa, J., Ishida, M., Nakajima, T., Honda, Y., Kitao, O., Nakai, H., Vreven, T., Montgomery, Jr., J. A., Peralta, J. E., Ogliaro, F., Bearpark, M., Heyd, J. J., Brothers, E., Kudin, K. N., Staroverov, V. N., Keith, T., Kobayashi, R., Normand, J., Raghavachari, K., Rendell, A., Burant, J. C., Iyengar, S. S., Tomasi, J., Cossi, M., Rega, N., Millam, J. M., Klene, M., Knox, J. E., Cross, J. B., Bakken, V., Adamo, C., Jaramillo, J., Gomperts, R., Stratmann, R., E., Yazyev, O., Austin, A. J., Cammi, R., Pomelli, C., Ochterski, J. W., Martin, R. L., Morokuma, K., Zakrzewski, V. G., Voth, G. A., Salvador, P., Dannenberg, J. J., Dapprich, S., Daniels, A. D., Farkas, O., Foresman, J. B., Ortiz, J. V., Cioslowski, J. & Fox, D. J. In *Gaussian 09 (Revision D.01)*, Gaussian, Inc.: Wallingford CT (2013).
7. Perdew, J. P., Burke, K. & Ernzerhof, M. Generalized gradient approximation made simple. *Phys. Rev. Lett.* **77**, 3865−3868 (1996).
8. Perdew, J. P., Burke, K. & Ernzerhof, M. Errata: generalized gradient approximation made simple. *Phys. Rev. Lett.* **78**, 1396 (1997).
9. Adamo, C. & Barone, V. Toward reliable density functional methods without adjustable parameters: the PBE0 model. *J. Chem. Phys.* **110**, 6158−6170 (1999).
10. Barone, V. & Cossi, M. Quantum calculation of molecular energies and energy gradients in solution by a conductor solvent model. *J. Phys. Chem. A* **102**, 1995−2001 (1998).
11. Cossi, M., Rega, N., Scalmani, G. & Barone, V. Energies, structures, and electronic properties of molecules in solution with the C-PCM solvation model. *J. Comput. Chem.* **24**, 669−681 (2003).
12. Andrae, D., Häuβermann, U., Dolg, M., Stoll, H. & Preuβ, H. Energy-adjustedab initio pseudopotentials for the second and third row transition elements. *Theor. Chim. Acta.* **77**, 123−141 (1990).

13. Ehlers, A. W., Böhme, M., Dapprich, S., Gobbi, A., Höllwarth, A., Jonas, V. Köhler, K. F., Stegmann, R., Veldkamp, A. & Frenking, G. A set of *f*-polarization functions for pseudo-potential basis sets of the transition metals Sc−Cu, Y−Ag and La−Au. *Chem. Phys. Lett.* **208**, 111−114 (1993).
14. Hehre, W. J., Ditchfie. R. & Pople, J. A. Self−Consistent molecular orbital methods. XII. Further extensions of Gaussian−type basis sets for use in molecular orbital studies of organic molecules *J. Chem. Phys.* **56**, 2257−2261 (1972).
15. Hariharan, P. C. & Pople, J. A. The influence of polarization functions on molecular orbital hydrogenation energies. *Theor. Chim. Acta.* **28**, 213−222 (1973).
16. Francl, M. M., Pietro, W. J., Hehre, W. J., Binkley, J. S., Gordon, M. S., Defrees, D. J. & Pople, J. A. Self-consistent molecular orbital methods. XXIII. A polarization-type basis set for second-row elements. *J. Chem. Phys.* **77**, 3654−3665 (1982).
17. te Velde, G., Bickelhaupt, F. M., Baerends, E. J., Guerra, C. F., Van Gisbergen, S. J. A., Snijders, J. G. & Ziegler, T. Chemistry with ADF. *J. Comput. Chem.* **22**, 931−967 (2001).
18. Guerra, C. F., Snijders, J. G., te Velde, G. & Baerends, E. J. Towards an order-N DFT method. *Theor. Chem. Acc.* **99**, 391−403 (1998).
19. Wu, N. M.-W., Ng, M. & Yam, V. W.-W. Photochromic benzo[*b*]phosphole alkynylgold(I) complexes with mechanochromic property to serve as multistimuli-responsive materials. *Angew. Chem. Int. Ed.* **58**, 3027−3031 (2019).
